# The 103,200-arm acceleration dataset in the UK Biobank revealed a landscape of human sleep phenotypes

**DOI:** 10.1073/pnas.2116729119

**Published:** 2022-03-18

**Authors:** Machiko Katori, Shoi Shi, Koji L. Ode, Yasuhiro Tomita, Hiroki R. Ueda

**Affiliations:** ^a^Department of Information Physics and Computing, Graduate School of Information Science and Technology, The University of Tokyo, Tokyo 113-0033, Japan;; ^b^Department of Systems Pharmacology, Graduate School of Medicine, The University of Tokyo, Tokyo 113-0033, Japan;; ^c^Laboratory for Synthetic Biology, RIKEN Center for Biosystems Dynamics Research, Osaka 565-5241, Japan; and; ^d^Sleep Center, Toranomon Hospital, Tokyo 105-8470, Japan

**Keywords:** sleep, sleep landscape, clustering, UMAP, insomnia

## Abstract

Human sleep phenotypes can be defined and diversified by both genetic and environmental factors. However, some sleep phenotypes are difficult to evaluate without long-term, precise sleep monitoring, for which simple yet accurate sleep measurement is required. To solve this problem, we recently developed a state-of-the-art sleep/wake classification algorithm based on wristband-type accelerometers, termed ACCEL (acceleration-based classification and estimation of long-term sleep-wake cycles). In this study, we optimized and applied ACCEL to large-scale analysis of human sleep phenotypes. The clustering of an about 100,000-arm acceleration dataset in the UK Biobank using uniform manifold approximation and projection (UMAP) dimension reduction and density-based spatial clustering of applications with noise (DBSCAN) clustering methods identified 16 sleep phenotypes, including those related to social jet lag, chronotypes (“morning/night person”), and seven different insomnia-like phenotypes. Considering the complex relationship between sleep disorders and other psychiatric disorders, these unbiased and comprehensive analyses of sleep phenotypes in humans will not only contribute to the advancement of biomedical research on genetic and environmental factors underlying human sleep patterns but also, allow for the development of better digital biomarkers for psychiatric disorders.

## Significance

Human sleep phenotypes are diversified by genetic and environmental factors, and a quantitative classification of sleep phenotypes would lead to the advancement of biomedical mechanisms underlying human sleep diversity. To achieve that, a pipeline of data analysis, including a state-of-the-art sleep/wake classification algorithm, the uniform manifold approximation and projection (UMAP) dimension reduction method, and the density-based spatial clustering of applications with noise (DBSCAN) clustering method, was applied to the 100,000-arm acceleration dataset. This revealed 16 clusters, including seven different insomnia-like phenotypes. This kind of quantitative pipeline of sleep analysis is expected to promote data-based diagnosis of sleep disorders and psychiatric disorders that tend to be complicated by sleep disorders.

Scientific and technological advancements often provide a landscape of our world and shape new ways to understand it. In the life sciences over the past 20 y, from the analysis of the whole human genome to the development of next generation sequencing, the landscape of genome science has changed dramatically (1–4). In fact, there have been many sustained efforts to understand the genetic landscape of diseases such as cancer, neurodegenerative diseases, and psychiatric disorders by conducting large-scale genome-wide association studies and comprehensive analyses of genetic variants (5, 6). In recent years, the trend of big data analysis has been moving toward a future where in-depth genetic analysis is combined with rich phenotype analysis (7). In this context, large-scale analysis of human phenotypes that considers both genetic as well as environmental factors will be important for gaining a deeper understanding of the topics of investigation.

Sleep is a physiological phenomenon that is widely conserved throughout the animal kingdom. Its basic structure is genetically conserved within species—humans are no exception to this. However, this structure can change either transiently or chronically depending on environmental factors (8). This effect of environmental factors on sleep can be observed in terms of the diversification of modern lifestyles. Humans are basically diurnal animals that are active during the day and sleep at night, but the widespread use of electricity has freed us from this constraint. It is also possible to temporarily affect wakefulness or sleep, as a way to resist genetically predetermined sleep times, through the intake of substances such as caffeine or alcohol. The way we work has also diversified as shift work and other types of work schedules have become more popular (8, 9). However, there are health trade-offs to this diversification of sleep schedules. For example, a night person tends to have less sleep on weekdays (10, 11), probably due to the social obligation of attending school or work in the morning. This might be associated with poorer concentration and academic/professional performance in the morning in such individuals (12). This leads to the concept of “social jet lag,” representing the difference in sleep duration between weekdays and weekends or holidays, which is a concern due to the potential for adverse health effects (13, 14). The diverse sleep phenotypes are not independent of mortality risks from chronic diseases such as cardiovascular disease, metabolic syndrome, and diabetes, although the complex relationships between them remain poorly understood (15).

The major effects of sleep shortage include physical effects (drowsiness, fatigue, and high blood pressure), cognitive impairment (decreased performance, attention, motivation, mental focus, and intellectual capacity), immune system dysfunction, and possible complications related to mental illnesses (16); 60 to 70% of adults are known to have sleep-related anxiety/problems, some of which are defined as sleep disorders (17). For example, hypersomnia is characterized by significant daytime sleepiness despite sufficient sleep at night (18). Circadian rhythm sleep disorder is another well-known sleep disorder and is characterized by a discrepancy between the actual sleep schedule and the required sleep schedule. One of the major sleep disorders is insomnia, which presents as a combination of the symptoms (difficulty in initiating sleep, difficulty in maintaining sleep, and waking up earlier than necessary) and associated daytime consequences. About 20% of the general population has insomnia symptoms three or more nights a week, of which about half have daytime consequences and meet the criteria of an insomnia diagnosis (19).

Several instruments are available to assist health care providers in evaluating complaints of insomnia. Polysomnography (PSG), which monitors such as brain activity and muscle movements, is the gold standard for evaluating sleep disorders such as insomnia, hypersomnia, and sleep apnea, and it is essential for the definitive diagnosis of these disorders. Since long-term measurements using PSG are not feasible, sleep questionnaires are also used with PSG measurement for diagnosis today (20, 21). However, these questionnaire-based estimates do not reflect the absolute values obtained by PSG. Thus, wristband accelerometers are commonly used as an alternative to PSG and sleep questionnaires. In fact, they have been used for many large-scale analyses so far (22–24) and have led to the discovery of several genes involved in sleep time regulation (25, 26). In combination with machine learning, it is now possible to capture the detailed structure of sleep, including midawake. The large-scale dataset thus obtained enabled us to systematically classify and interpret the various sleep phenotypes in modern society at a high resolution. Ultimately, large-scale measurements and automatic and quantitative classification of sleep phenotypes could reduce the workload of clinicians and may even lead to the discovery of rare phenotypes that have not received much attention so far.

**Fig. 1.**
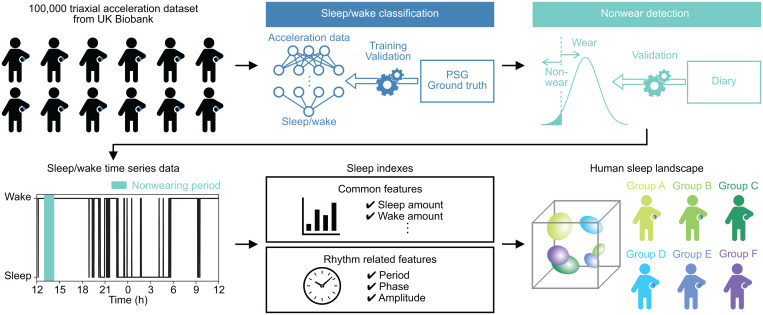
Overview. About 100,000 triaxial acceleration datasets stored in the UK Biobank were converted to the sleep/wake time series data through the sleep/wake classification and the nonwear detection algorithms. The sleep/wake time series data were then converted to 21 sleep indexes. Lastly, the landscape of human sleep phenotypes was classified by clustering methods based on the sleep indexes.

In this study, as a first effort at automatic classification of sleep phenotypes, we classified sleep phenotypes based on over 100,000 accelerometer–acquired datasets in the UK Biobank (27). The large-scale acceleration data were converted to sleep/wake time series data by combining the state-of-the-art sleep/wake classification algorithm, termed ACCEL (28), and a nonwear detection algorithm. We calculated 21 sleep indexes from sleep/wake time series data and applied manifold-based dimension reduction and clustering methods (29, 30). The systematic and unsupervised clustering of the large-scale dataset revealed 16 clusters, representing distinctive sleep phenotypes that are consistent with medically described conditions, including social jet lag–related sleep phenotypes and several insomnia-related sleep phenotypes (Fig. 1).

## Results

### Sleep/Wake Time Series Data.

In this study, we further optimized and applied the algorithm of ACCEL to extract sleep/wake time series data from acceleration data obtained using Axivity, the activity-tracking wristband with a triaxial accelerometer used in the UK Biobank project (27). The original sleep/wake classification algorithm is a machine learning–based algorithm that uses XGBoost and the power spectrum of jerk (a derivative of acceleration) as its features (28). In this study, we simultaneously acquired 27 PSG data and Axivity acceleration data and optimized the sleep/wake classification algorithm for Axivity (*SI Appendix*, Table S1). The Axivity signal had lower amplitudes in terms of jerk and the power spectrum during sleep epochs than wake epochs, as was shown in a previous study (*SI Appendix*, Fig. S1 *A–C*) (28). The XGBoost hyperparameters were optimized to maximize the summation of accuracy and F measure the sleep/wake classification algorithm using Bayesian optimization (*SI Appendix*, Fig. S1*D*). We also adopted the nonwear detection algorithm from a previous study (31); this algorithm uses the thresholds of two features, SD and range of acceleration, to predict nonwearing periods. In this study, we analyzed the distributions of these features between wearing and nonwearing periods and demonstrated that the thresholds proposed in the previous study are adaptable to Axivity (*SI Appendix*, Fig. S1*E* and *F* and Table S2). As a result of combining the sleep/wake classification and nonwear detection algorithms, the algorithm used in this study achieved high sensitivity (97.20 ± 2.38%) and specificity (82.19 ± 12.03%); these are the percentages that the algorithm correctly classified sleep epochs as sleep and wake epochs as wake, respectively (*SI Appendix*, Fig. S1*G*). We also confirmed that our algorithm shows high performance for acceleration data with a different sampling frequency (*SI Appendix*, Fig. S1 *H* and *I* and Table S3). Moreover, the high specificity of the algorithm allowed us to accurately detect short-term awake episodes during sleep, which had been difficult in previous studies (22–24). We also calculated two standard sleep indexes, total sleep time (TST), and wake after sleep onset (WASO). These are used to characterize sleep structures in PSG-based studies and are also often used to evaluate the performance of sleep/wake classification algorithms (*SI Appendix*, Fig. S2 *A–D*) (22–24). In this study, sleep onset and offset were defined from sleep/wake time series data, during which summation of sleep time and awaking time were measured as TST and WASO. Bland–Altman plots show that our algorithm overestimated TST and WASO by only 5.89 and 1.43 min, respectively (*SI Appendix*, Fig. S2 *E* and *F*), which are almost comparable with other previous studies (22–24).

### Sleep Indexes Extraction.

**Fig. 2.**
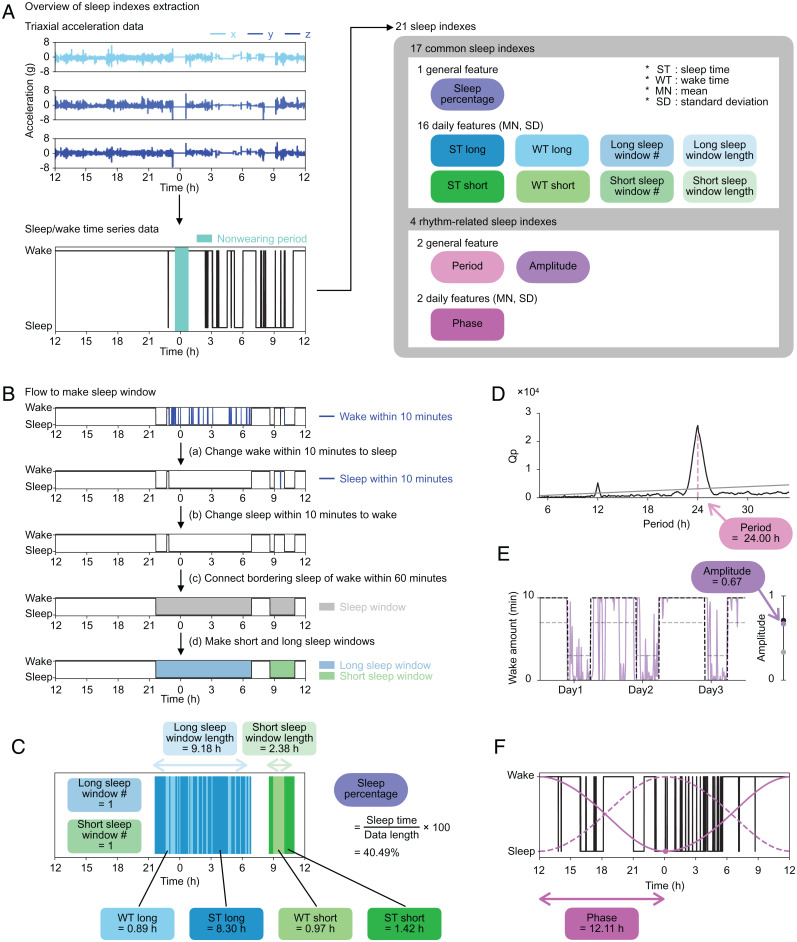
Sleep index extraction. (*A*) The overview of sleep indexes extraction. Each set of axial data is shown in the three panels in *Upper Left* (row 1: *x*; row 2: *y*; row 3: *z*). The sleep/wake time series data are shown in the same format as in Fig. 1. Twenty-one sleep indexes converted from the sleep/wake time series data, including 17 common sleep indexes and four rhythm-related sleep indexes. The sleep indexes, calculated as a single value throughout the measurement period, were named general features (oval icons). From the daily features (rectangle icons), both MN and SD were included in the sleep indexes. (*B*) The procedures to make the sleep window. We changed epochs of continuous wake or sleep for less than 10 min to sleep or wake, respectively. The sleep window was created by connecting sleep epochs, ignoring waking epochs of 60 min or less. Long sleep windows (blue) and short sleep windows (green) are made based on the length of the sleep window. (*C*) An example of noon-to-noon data and common sleep indexes calculated for a day. (*D*) The result of the chi-square periodogram. The black line shows the Qp values (a statistic of chi-square), and the gray line shows 0.01 levels of statistical significance ranging from 5.00 to 35.00 h. The pink dashed line shows the point when the difference between Qp and the significant value is at its maximum, and its value, in this case 24.00 h, is used as the period. (*E*) The purple line shows wake amount per 10 min. The black and gray dashed lines represent a 24-h periodic square wave signal with 1/3 duty in the range from 0 to 10 min and from 3 to 7 min, respectively. The dots on the right bar show the amplitudes of the three lines that are calculated as the coefficient of variation SD/mean. The purple dot plotted at 0.67 is the amplitude of this example data. (*F*) The black line shows the sleep/wake time series data. The dashed and solid magenta lines are the van der Pol limit cycles. The dashed line is the curve with the minimum point at noon. The solid line is a fitted curve to the sleep/wake time series data, and the dot is the minimum point of this curve. The duration between the minimum point and the last noon is calculated as the phase: in this case, 12.11 h.

Generally, people take the longest sleep at night, but due to the increased diversity of social life, some people sleep longest during the day (32). In addition, variation in total sleep amount per day, high WASO, and low TST are sometimes considered as hallmarks of sleep disorders (33). To capture the diverse structures of sleep, we converted sleep/wake time series data to a total of 21 sleep indexes, including 17 common sleep indexes representing quantity-related features and four rhythm-related sleep indexes representing circadian rhythm–related features (Fig. 2*A* and *SI Appendix*, Table S4).

The 17 common sleep indexes include the number and length of sleep windows, which represent the time zone of dense sleep (34), and sleep time (ST) and wake time (WT), which represent sleep duration and midawake duration during sleep windows, respectively. In this study, two different sleep windows were considered to capture both long and short sleep windows according to the following procedure (Fig. 2*B*). Convert wake periods of less than 10 min to sleep periods and vice versa (Fig. 2 *B*, *a*and *b*). The threshold of 10 min was determined by verifying the sensitivity and specificity of the sleep/wake classification algorithm using the pseudo-sleep/wake time series data (*SI Appendix*, Fig. S3 *A–C*). Evaluate the length of time gaps between sleep periods. If the duration is less than 60 min, connect them as a sleep window (35) (Fig. 2 *B*, *c*). According to the length of each sleep window, name it a short sleep window if shorter than a threshold or a long sleep window if longer than a threshold (Fig. 2 *B*, *d* and *SI Appendix*). Fig. 2*C* shows an example of sleep data of 1 day from noon to the next noon (noon-to-noon data). There is one long sleep window and one short sleep window, where the threshold was set as 3.75 h. The blue area in Fig. 2*C* shows the long sleep window from after 9:00 PM until about 7:00 AM, including a total of 0.89 h of wake episodes (WT long) and 8.30 h of sleep episodes (ST long). The green area in Fig. 2*C* shows that there is a short sleep window from before 9:00 AM until about 11:00 AM, during which there is 0.97 h of wake episodes (WT short) and 1.42 h of sleep episodes (ST short). The percentage of sleep time in 24 h (sleep percentage) is 40.49%. For subjects with multiple days of measurement, mean (MN) and SD were calculated for each sleep index.

The rhythm-related sleep indexes adopted in this study (period, amplitude, and phase) are commonly used features in the field of circadian rhythm research (Fig. 2 *D–F*) (36–39). Period was calculated as the maximum peak of chi-square periodogram (*SI Appendix*, Fig. S3 *D–F*) (36). It is usually difficult to obtain the true amplitude of circadian rhythms without various perturbations (such as light illumination). Therefore, amplitude in this study was defined as a coefficient of variation (SD/mean) of wake amount per 10 min, as in a previous study (Fig. 2*E*) (37), to represent the amplitude of a circadian output to sleep. In the case of data in Fig. 2 *D* and *E*, period is 24.00 h, and amplitude is 0.67. To calculate phase, we used the van der Pol limit cycle, which is a classical model for calculating the circadian phase in human studies (38, 39). After fitting data, we defined phase as the duration between the minimum point on the fitted curve (the magenta point in Fig. 2*F*) and the last noon. In the case of data in Fig. 2*F*, phase is 12.11 h.

### Distribution of Sleep Indexes.

**Fig. 3.**
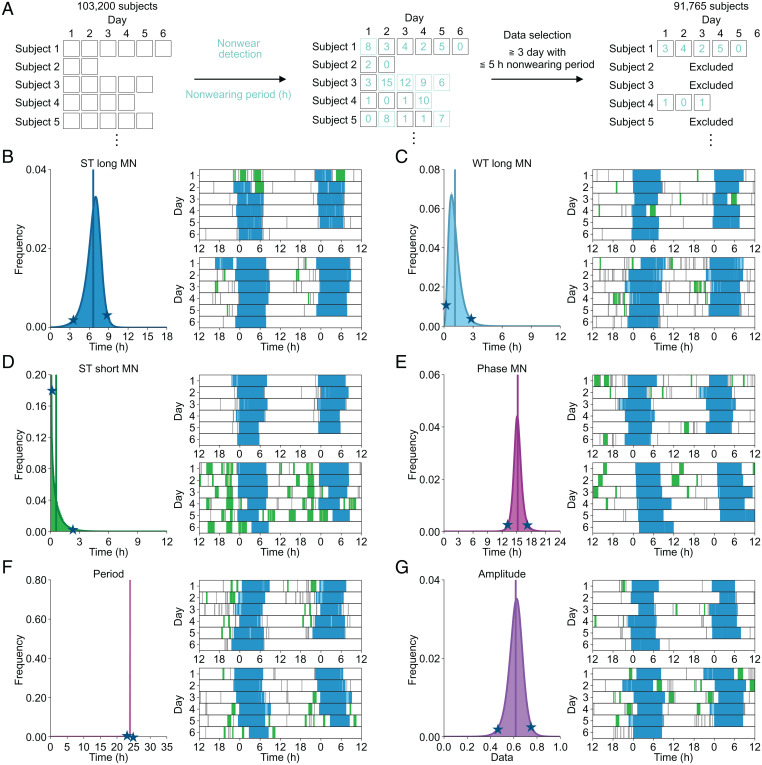
Distribution of sleep indexes. (*A*) The flow of data exclusion for large-scale sleep analysis. Nonwearing periods (emerald green) were calculated for noon-to-noon data. The noon-to-noon data with less than 5 h of nonwearing period and continuing more than 3 d were used for the large-scale sleep analysis (black squares). In this schema, data 1 and 4 are included in the large-scale sleep analysis. (*B–G*), *Left* shows the distribution of sleep index, with the mean and the fitted curve shown as the solid lines and solid curves, respectively. The stars show the locations of representative plots (lower or upper 2.28 percentiles) shown as double plots in *Right*, where ST long, WT long, ST short, and WT short are colored the same color as the icons of sleep indexes in Fig. 2*A*. The sleep epochs outside long and short sleep windows are shown in gray.

In this study, we analyzed the Axivity dataset of 103,200 subjects over 522,826 d in total, with up to 7 d of continuous measurement from the UK Biobank project (27). To calculate amplitude and period, we selected individual records from subjects with more than 3 d of continuous measurement and less than 5 h of nonwearing period (Fig. 3*A* and *SI Appendix*, Fig. S4*A*); 91,765 subjects met these criteria, and their acceleration data were converted into sleep/wake time series data for the calculation of 21 sleep indexes. The threshold between the long and short sleep windows was determined by setting a threshold based on the distribution of sleep window length (*SI Appendix*, Fig. S4*B*).

The correlation coefficients between the sleep indexes are shown in *SI Appendix*, Fig. S4*C*; the median of coefficients is 0.09, which indicates that there are weak correlations between the sleep indexes. We plotted distributions for each sleep index (Fig. 3 *B–G* and *SI Appendix*, Fig. S4*D–R*), where curve fitting was conducted for all distributions, except for integral and SD features, to capture their shapes. The mean of ST long MN was 6.60 h (Fig. 3*B*) (age = 62.01 ± 7.82). When we analyzed the data for subjects in their 70s, the mean of ST long MN was 6.33 h, which is close to TST in a PSG-based large-scale study (*n* = 889, age = 76.28 ± 5.47) (40). The representative plots from both sides (2.28 percentile) of distribution are shown in Fig. 3 *B–G*. We found that the representative plots where certain sleep indexes are in the upper or lower 2.28 percentiles show interesting sleep phenotypes (Fig. 3 *C–E*), some of which are similar to sleep disorder–like phenotypes. With regard to the representative plot with high WT long MN shown in Fig. 3*C*, awake time during the long sleep window per day is 170.25 min, which may indicate an insomnia-like sleep phenotype with long WASO (33). Similarly, the representative plot with high ST short MN shown in Fig. 3*D* includes longer daytime sleep episodes (137.83 min/d), which are a feature of hypersomnia (33). The mean of phase MN was 15.22 h, where representative plots with low and high phase represent phenotypes of a morning person (subjective midnight at around 1:30 AM) and a night person (subjective midnight at around 5:30 AM), respectively (Fig. 3*E*). Thus, it may be possible to find a sleep phenotype that has the same characteristics as a particular sleep disorder by setting a proper threshold; however, setting a threshold can sometimes be arbitrary. In addition, the correlations among 21 sleep indexes (*SI Appendix*, Fig. S4*C*) could make classification difficult. Therefore, we decided to use all 21 sleep indexes for our classification.

### Dimension Reduction and Clustering.

**Fig. 4.**
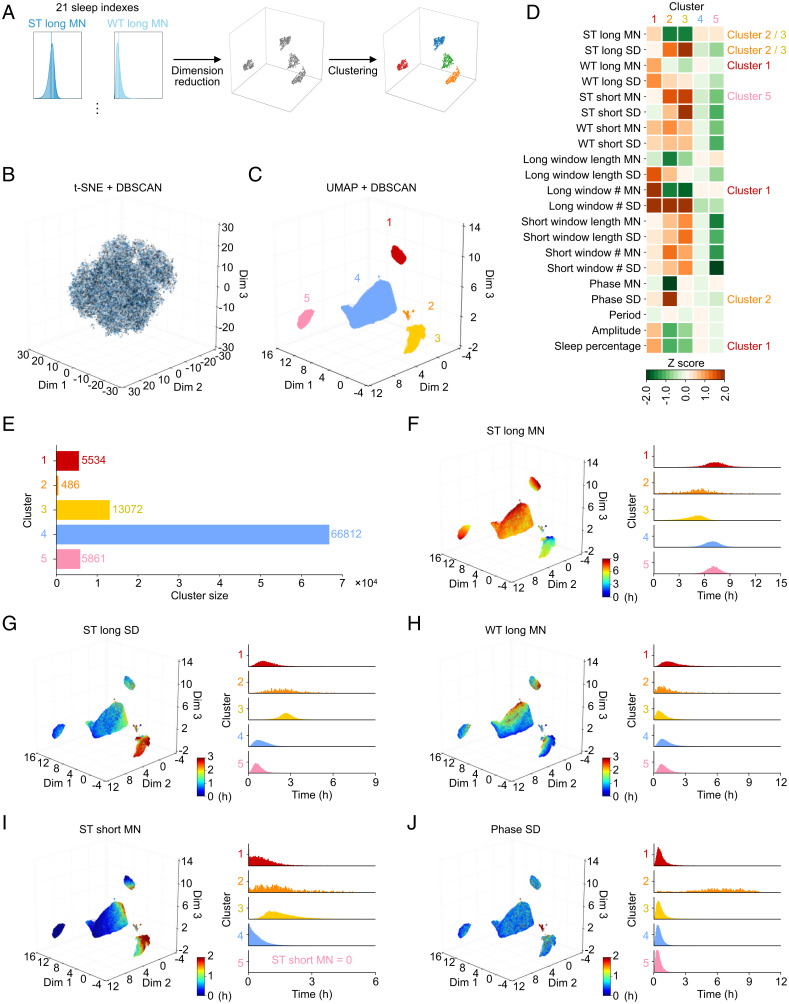
Clustering analysis revealed five clusters. (*A*) The flow of clustering. (*B*) The result of t-SNE and DBSCAN. Individual records are divided into many small clusters. (*C*) The result of UMAP and DBSCAN. Datasets are divided into five clusters named clusters 1 to 5. (*D*) Heat map of *z* score. The names of clusters are shown next to their main features. (*E*) The size of each cluster. The histogram is colored using the same colors as in *C*. (*F–J*), *Left* represents the result of first clustering, where each individual record is colored corresponding to the heatmap of each sleep index. *Right* shows the histograms of the distribution of each sleep index. The scale of the *y* axis is the same among clusters and was set based on the range of histogram values. The histogram is colored with the same colors as in *C*.

For analyses of large-scale data with multiple dimensions, dimension reduction methods usually play an important role and enable us to visualize high-dimensional data in a two- or three-dimensional space. In this study, dimension reduction methods were applied to 21 sleep indexes to classify sleep phenotypes and delineate the sleep landscape (Fig. 4*A*). We applied four methods—principal component analysis (PCA), t-distributed stochastic neighbor embedding (t-SNE), uniform manifold approximation and projection (UMAP), and a combination of PCA and UMAP—to the 21-dimensional data and converted them to 3-dimensional data, resulting in UMAP dividing the dataset into more interpretable clusters than the other methods (Fig. 4 *B* and *C* and *SI Appendix*, Figs. S5 *A–D* and S6*A*). Therefore, we selected the UMAP method in this study and further applied a clustering method known as density-based spatial clustering of applications with noise (DBSCAN).

As a result, we obtained five clusters (clusters 1 to 5) as shown in Fig. 4*C*. The features and population size of each cluster are shown in Fig. 4 *D* and *F–J* and *SI Appendix*, Fig. S5 *E–T* and in Fig. 4*E*, respectively. Cluster 1 has higher WT long MN than other clusters (Fig. 4 *D* and *H*), with more than one long sleep window per day (*SI Appendix*,Fig. S5*K*) and thus, high sleep percentage (*SI Appendix*, Fig. S5*T*), which means that the subjects in cluster 1 slept more and with long-term midawake. Since difficulty in maintaining sleep is one of major characteristics of insomnia (18, 33), we named cluster 1 “insomnia with long sleep duration and midawake.” The criteria for an insomnia diagnosis include both nocturnal sleep problems and daytime consequences, but the UK Biobank dataset used in this study does not have additional information about daytime complaints, which led to the focus being on only one aspect of insomnia, nocturnal sleep problems. Both clusters 2 and 3 are characterized by low ST long MN and high ST long SD (Fig. 4 *D*, *F*, and *G*), while their phase SDs are different (Fig. 4 *D* and *J*). High phase SD in cluster 2 might capture an irregular feature of subjects’ lifestyle. Another feature of cluster 2 is that ST long MN varies between days (Fig. 4*G*), suggesting that the length and timing of sleep for subjects in cluster 2 vary from day to day. Meanwhile, cluster 3 shows normal phase SD (Fig. 4*J*) and high ST long SD (Fig. 4*G*), suggesting that the subjects in cluster 3 slept at almost the same time every day but that the sleep amount per day varied. Cluster 4 contains about 72.81% of total population (Fig. 4*E*), and its features are almost the same as those of whole datasets (Fig. 4*D*), suggesting that cluster 4 is the major cluster. Cluster 5 has zero ST short MN, while the other sleep indexes are normal, which means that the subjects in cluster 5 do not have any daytime sleep (Fig. 4 *D* and *I* and *SI Appendix*, Fig. S5*O*).

**Fig. 5.**
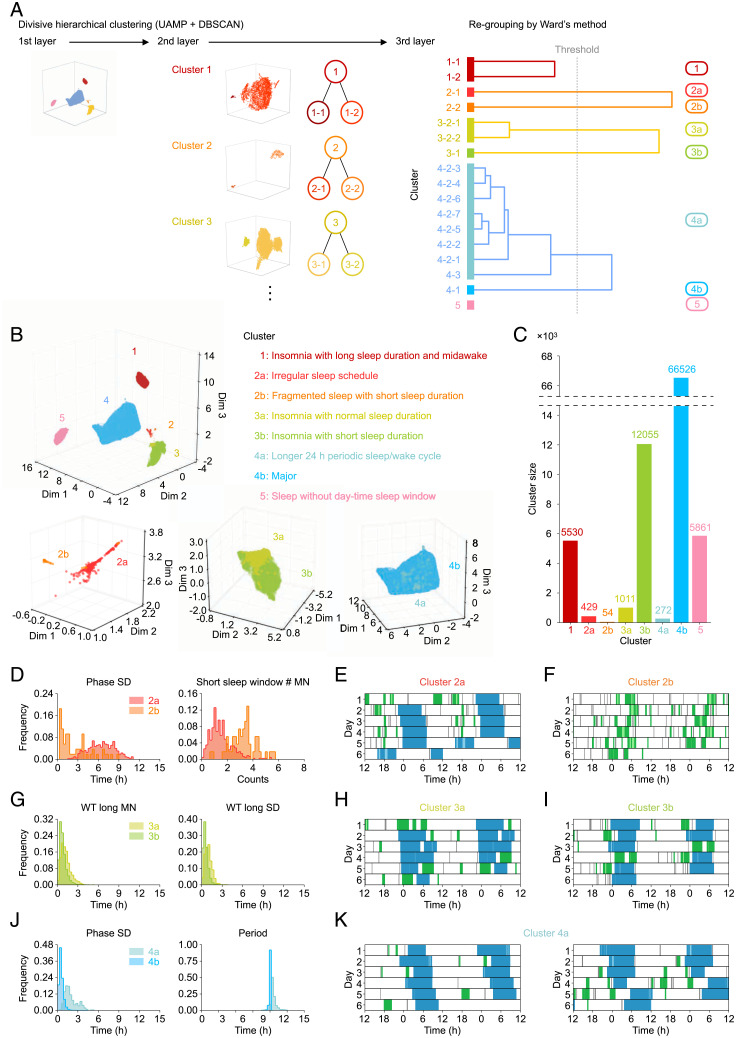
Hierarchical clustering analysis revealed eight clusters. (*A*) The flow of divisive hierarchical clustering. The same clustering process was repeated three times (*SI Appendix*). The 17 clusters obtained by divisive hierarchical clustering were regrouped using Ward’s method and named as clusters 1, 2a, 2b, 3a, 3b, 4a, 4b, and 5. (*B*) *Upper* shows the result of first-layer clustering, where each individual record was colored by clusters’ colors. The caption summarizes sleep phenotypes of each cluster. *Lower Left*, *Lower Center*, and *Lower Right* are the enlargement figures of clusters 2, 3, and 4, respectively. (*C*) The size of each cluster. Twenty-seven individual records were detected as noise by DBSCAN. (*D–K*) The distribution of sleep indexes of (*D*) clusters 2a and 2b, (*G*) clusters 3a and 3b, and (*J*) clusters 4a and 4b and representative plots of (*E*) cluster 2a, (*F*) cluster 2b, (*H*) cluster 3a, (*I*) cluster 3b, and (*K*) cluster 4a shown as double plots.

We obtained five clusters related to insomnia or lifestyle. Interestingly, the sleep indexes in some clusters suggested that they could be divided even further. For example, the distribution of phase SD of cluster 2 was broad, raising a possibility that it contains more than two clusters with different phase SD features (Fig. 4*J*). Therefore, we repeated the same clustering process (i.e., dimension reduction and clustering) for the individual records contained in each cluster and obtained the next-layer clusters (Fig. 5*A*). By repeating this clustering process until a cluster is not divided into at least two clusters with their size equaling 20 or larger, we obtained 17 clusters (*SI Appendix*, Tables S5 and S6). However, there were strong correlations between the 17 clusters, suggesting that some of the clusters may be easier to understand if they were regrouped together (*SI Appendix*, Fig. S6*B*). Thus, we regrouped 17 clusters by applying Ward’s method, an objective hierarchical clustering method, to their sleep indexes; regrouping across clusters 1 to 5 was not performed to maintain the first-layer relationship (Fig. 5*A* and *SI Appendix*, Fig. S6*B*). As a result, we obtained eight clusters, where clusters 1 and 5 were undivided, and clusters 2, 3, and 4 were divided into two subgroups (cluster 2a/b, cluster 3a/b, and cluster 4a/b) (Fig. 5 *B* and *C* and *SI Appendix*, Figs. S7–S9 and Tables S7 and S8).

Fig. 5*B* shows the eight clusters mapping on the space of clusters 1 to 5, where clusters 2a and 2b are spatially separated but the boundaries of clusters 3a, 3b, 4a, and 4b are not obvious. Cluster 2a contains about 88.27% of individual records in cluster 2 (Figs. 4*E* and 5*C*) and shows high phase SD (Fig. 5*D*), low ST long MN (*SI Appendix*, Fig. S7*A*), and high ST long SD (*SI Appendix*, Fig. S7*B*), as does cluster 2. Therefore, we named cluster 2a “irregular sleep schedule” (Fig. 5*E* and *SI Appendix*, Fig. S8*B*). This irregular sleep phenotype may be related to the difference in sleep/wake patterns between workdays and holidays or social jet lag (13). This sleep phenotype sometimes appears in rotating shift workers who alternate daytime and night shifts (9, 41). On the other hand, cluster 2b has no long sleep window (*SI Appendix*, Fig. S7*K*) but many short sleep windows, which lead to various phases during days and low sleep percentage (Fig. 5*D* and *SI Appendix*, Fig. S7*U*). These characteristics show that the subjects in cluster 2b repeated short-term sleep (Fig. 5*F* and *SI Appendix*, Fig. S8*C*), and thus, this cluster was named “fragmented sleep with short sleep duration.” Cluster 3 was divided into clusters 3a and 3b, where almost all features of cluster 3 are preserved (e.g., low ST long MN, high ST long SD, and constant phase SD). However, cluster 3a has higher WT long MN, WT long SD, and sleep percentage than cluster 3b (Fig. 5*G* and *SI Appendix*, Fig. S7*U*), which means that there are differences in the length of midawake and sleep duration between clusters 3a and 3b (Fig. 5 *H* and *I* and *SI Appendix*, Fig. S8 *D* and *E*). Thus, we named clusters 3a and 3b “insomnia with normal sleep duration” and “insomnia with short sleep duration,” respectively. Cluster 4 was divided into clusters 4a and 4b, with the distribution of period varying between them (Fig. 5*J*). Cluster 4a has a sleep/wake cycle with period apparently longer than 24 h (the mean of the period is 25.19 h), which leads to higher phase SD (Fig. 5*J*). Thus, we characterized cluster 4a as “longer 24-h periodic sleep/wake cycle,” where subjects were also identified by setting a threshold on the distribution of period (Fig. 3*F*). Notably, this apparent long period may represent a mixture of two different sleep phenotypes (Fig. 5*K*). Longer-term measurements (e.g., more than 2 wk) and more information about the subjects would allow for deeper insights. Cluster 4b is the largest group (Fig. 5*C*), and its distribution is close to that of the whole dataset, indicating that cluster 4b is the major group.

In summary, we identified eight clusters whose sleep indexes distribute smoothly (*SI Appendix*, Fig. S7), indicating that these clusters could not have been obtained by using only a threshold-based method but were revealed by using an unsupervised and unbiased clustering method like UMAP. On the other hand, groups like cluster 4a could be obtained by setting a proper threshold for the distribution of one sleep index (Fig. 3*F*), which suggests that some classifications could be revealed by using a threshold. In other words, it may be possible to understand human sleep phenotypes at a higher resolution by combining threshold-based and UMAP-based classification, especially in the understanding of sleep disorders. Although we have obtained a couple of insomnia-like clusters (Fig. 5*B*), this may be only a piece of sleep disorders, and larger clusters, like clusters 3b and 4b, may contain other small groups representing sleep disorders. Thus, if we can classify normal sleep and abnormal sleep based on some thresholds and then apply UMAP to abnormal sleep, we would obtain classifications of abnormal sleep at a higher resolution, which are expected to be related to sleep disorders.

### Outliers.

**Fig. 6.**
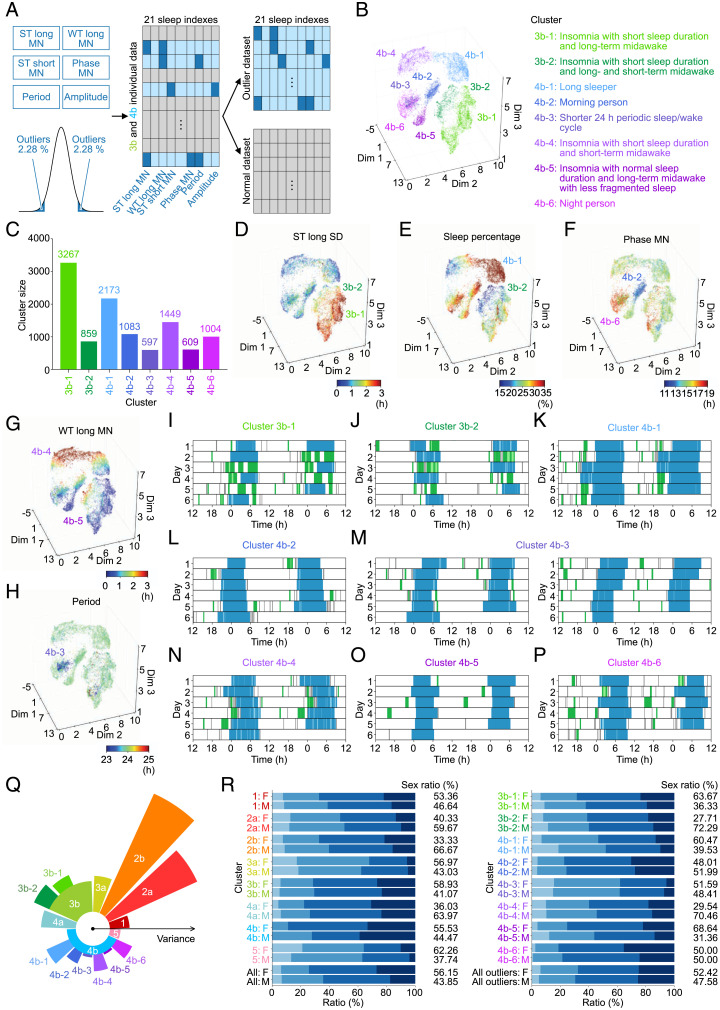
Clustering analysis of the outlier dataset revealed eight clusters. (*A*) The flow of data selection for the outlier clustering. Blue marks the lower and upper 2.28 percentiles in six sleep indexes in *Center*. The individual records with such values colored sky blue are divided as the outlier dataset, while the remaining individual records colored gray are divided as the normal dataset. (*B*) The result of clustering. The outlier dataset is divided into eight clusters. (*C*) The size of each cluster. Four hundred fifty-eight individual records were detected as noise by DBSCAN. (*D–H*) The results of outlier clustering, where each individual record is colored corresponding to the heatmap of each sleep index. (*I–P*) Representative plots of clusters in the outlier clustering shown as double plots. (*Q*) The summary of whole clustering and outlier clustering. The radius of each cluster shows the L2 norm between the mean of each cluster and that of whole dataset (the black center point). (*R*) Sex and age proportions of whole clustering and outlier clustering.

We chose six sleep indexes and set their lower or upper 2.28 percentiles as an outlier dataset in clusters 3b and 4b (Fig. 6*A*). The proportions of the outlier data in clusters 3b and 4b were 35.63 and 10.83%, respectively. These discretized datasets were further classified by using UMAP and DBSCAN, which revealed eight clusters; of these, two (clusters 3b-1 and 3b-2) were derived from cluster 3b, and the others (clusters 4b-1 to 4b-6) were derived from cluster 4b (Fig. 6 *B–P* and *SI Appendix*, Figs. S10–S12 and Tables S9 and S10). We confirmed that similar clusters, clusters b and c and clusters e to j, can be obtained when the analysis is applied to the entire outlier dataset, corresponding to clusters 3b-1 and 3b-2 (clusters b and c) and clusters 4b-1 to 4b-6 (clusters e to j), respectively (*SI Appendix*, Fig. S12 *E* and *F*). As shown in Fig. 6*B*, there are four more insomnia-like clusters (*SI Appendix*, Tables S11 and S12) and four more lifestyle-related clusters in these eight clusters.

Both clusters 3b-1 and 3b-2 have the insomnia-like phenotype characterized by a long sleep window divided into multiple short sleep windows just like cluster 3b (Fig. 6 *I* and *J* and *SI Appendix*, Fig. S11 *A* and *B*). Fig. 6*D* shows one feature of this type of insomnia-like phenotype, where clusters 3b-1 and 3b-2 have higher ST long SD (*SI Appendix*, Fig. S10*B*). Thus, clusters 3b-1 and 3b-2 can be considered as subtypes of the insomnia-like phenotype in terms of different distributions of WT long MN from cluster 3b (Fig. 6*G* and *SI Appendix*, Fig. S10*C*). The subjects in cluster 3b-1 have long-term midawake at night while having low WT long MN. These features demonstrate that the subjects in cluster 3b-1 could not fall asleep smoothly after midawake, although they sleep with normal numbers of midawake (Fig. 6*I* and *SI Appendix*, Fig. S11*A*). On the other hand, cluster 3b-2 is characterized by higher WT long MN than cluster 3b and by both long-term and short-term midawake at night, which leads to insufficient sleep amount (Fig. 6*J* and *SI Appendix*, Figs. S10*U* and S11*B*). The phenotypes of clusters 4b-4 and 4b-5 are also subtypes of the insomnia-like phenotype, but sleep in both clusters is not fragmented as severely as in cluster 3–related clusters. The subjects in cluster 4b-4 had repeated short-term midawake at night (Fig. 6*N* and *SI Appendix*, Fig. S11*F*), which is reflected as high WT long MN (Fig. 6*G*) and leads to the insomnia-like phenotype with unfragmented and short sleep duration. In contrast, cluster 4b-5 is characterized by low WT long MN (Fig. 6*G*) and long-term midawake (Fig. 6*O* and *SI Appendix*, Fig. S11*G*), which are the same as cluster 3b-1 (Fig. 6*I* and *SI Appendix*, Fig. S11*A*). The difference between them is how fragmented their sleep is, in that sleep in cluster 4b-5 is fragmented less often.

Cluster 4b-1 is characterized by long sleep duration at night without long-term midawake, as indicated by high long sleep window length MN (*SI Appendix*, Fig. S10 *C* and *I*). Sleep percentage (Fig. 6*E* and *SI Appendix*, Fig. S10*U*) is high not because of multiple sleep windows but due to one long sleep window of about 9 h (Fig. 6*K* and *SI Appendix*, Fig. S11*C*), suggesting that the phenotype of cluster 4b-1 is of a long sleeper. Hypersomnia is characterized by long sleep and daytime naps. The subjects in cluster 4b-1 only have the former characteristic, suggesting that they have no complaints about daytime naps and do not fall in the standard categorization of hypersomnia (42). Clusters 4b-2 and 4b-6 stand out in terms of phase MN, where cluster 4b-2 has lower phase MN, while cluster 4b-6 has higher phase MN than the whole dataset (Fig. 6*F* and *SI Appendix*, Fig. S10*Q*), indicating that the subjects in clusters 4b-2 and 4b-6 are morning persons and night persons, respectively (Fig. 6 *L* and *P* and *SI Appendix*, Fig. S11 *D* and *H*). The last cluster, cluster 4b-3, shows apparent shorter 24-h periods (Fig. 6*H* and *SI Appendix*, Fig. S10*S*) in contrast to cluster 4a. However, there are two types of sleep phenotypes (Fig. 6*M*) like the two types included in cluster 4a.

In summary, the large-scale sleep phenotype analysis on acceleration data from over 100,000 subjects identified 16 clusters (Fig. 6*Q*). We also investigated if these 16 clusters are related to the season in which they were measured or the age of the subjects. The proportions of sex, age (40s, 50s, 60s, and 70s), and measurement month in each cluster are shown in Fig. 6*R* and *SI Appendix*, Fig. S12*G*. We found that there is no obvious correlation between the cluster and the measurement season or month. Notably, the proportion of subjects in their 70s in cluster 2b is higher than that in other clusters (Fig. 6*R*). As age increases, WASO has been reported to gradually increase, and TST has been reported to decrease, suggesting that some elderly people might have such a phenotype (43). Therefore, the 16 clusters demonstrate various sleep phenotypes and reveal a first draft landscape of human sleep phenotypes.

## Discussion

In this study, we conducted a large-scale systematic analysis of the diversity of sleep phenotypes using about 100,000 long-term acceleration data collected by the UK Biobank (Fig. 1). The acceleration data were converted to 21 sleep indexes using a state-of-the-art sleep/wake classification algorithm and a nonwear detection algorithm (Fig. 2*A*). By applying UMAP and DBSCAN, we obtained 16 clusters (clusters 1, 2a, 2b, 3a, 3b, 4a, 4b, and 5 and clusters 3b-1, 3b-2, and 4b-1 to 4b-6), including insomnia-like and lifestyle-related clusters (Figs. 5*B* and 6*B*). Parts of these insomnia-like clusters were identified by focusing on the outliers that have at least one sleep index with a value in the upper or lower 2.28 percentiles of distribution (Fig. 6*B*)—cluster 4b-4 represents an insomnia-like phenotype with unfragmented and short sleep duration, whereas cluster 4b-5 represents an insomnia-like phenotype with long-term midawake and less fragmented sleep. In this way, we identified several diverse sleep phenotypes and delineated a real-world sleep landscape (Fig. 6*Q*).

The irregular sleep schedule in cluster 2a involves a few days with delayed sleep onset compared with onset on other days, which might reflect different lifestyles on workdays and holidays. These delayed sleep schedules are also observed for rotating or fixed night shift workers, such as a sleep onset delay of about 7 h on night shift days compared with holidays and daytime workdays (9, 41), suggesting that cluster 2a could reflect the lifestyle of shift workers who work at night 1 or 2 d/wk. Most shift workers suffer from misalignments between the circadian rhythm and the required sleep schedule, which cause reduced sleep duration and low sleep quality after night work shifts (19, 44), often leading to depression and anxiety (16). Thus, quantitative detection of midawake and daytime sleep may be important in supporting the health of shift workers. Notably, shift workers represent about 16% of the working population (45), but cluster 2a accounts for only about 0.47% of the whole dataset (Fig. 5*C*), which may be due to the dataset used in this study being obtained over too short a period to identify shift work when the shifts are not very frequent. Measurements over a longer period (e.g., 2 wk) would be useful for more than just the identification of shift workers. Two clusters were identified based on the constant phase of sleep/wake cycle and named as “morning person” and “night person” (clusters 4b-2 and 4b-6, respectively). The question of whether the circadian clocks of these people or the phases shifted due to social constraints would be resolved by long-term measurements. If the shift is of the circadian clock, the subjects would have advanced sleep phase syndrome or delayed sleep phase syndrome, which are known to be linked to genetic factors (46, 47). Clusters 4a and 4b-3 are characterized by an apparent period that is shorter or longer than 24 h, while each of them includes two types of sleep phenotypes. In cluster 4a, for example, the sleep phenotype shown in Fig. 5 *K*, *Left* appears to be free running, while that shown in Fig. 5 *K*, *Right* can be considered as a difference between workdays (days 1 to 4) and holidays (days 5 and 6). In cluster 4b-3, Fig. 6 *M*, *Left* seems to have a short period, while the apparent short period of Fig. 6 *M*, *Right* is caused by the effects of working days; for example, days 1 to 3 were holidays followed by three continuous workdays. Long-term measurements may reveal the underlying environmental factors affecting these two phenotypes. Long circadian rhythms are observed in subjects with visual handicaps (48) or having lifestyles without sunlight exposure (49). Furthermore, using pseudo-sleep/wake time series data that included different sleep schedules per day, we verified that a 6-d measurement is not sufficient to completely distinguish between such sleep and short/long circadian rhythms (*SI Appendix*, Fig. S3*F*). Particularly, the period of such pseudo data tends to be underestimated, which is why such a short period cluster that is rarely observed in the light–dark cycle condition (48) appeared in this study. Of note, the pioneering large-scale study of human sleep succeeded in an extraction of several features corresponding to the diversity of human sleep phenotypes from long-term measurements (34). Winnebeck et al. (34) utilized acceleration data with more than weeks to extract time series features, which could be applied to our sleep landscape pipeline in the future. Advances in the machine learning field could drive and color the sleep landscape if we can use the sleep/wake time series data as the input instead of the sleep index. Thus, longer-term measurements and time series analyses can better identify the sleep phenotypes of subjects, which may further improve our understanding of genetic and environmental regulation of sleep.

The sleep/wake classification algorithm used in this study reached high specificity and sensitivity, demonstrating the ability to capture short-term midawake. This advantage led to identifying insomnia-like phenotypes characterized by midawake, known as difficulty in maintaining sleep. Seven clusters related to insomnia were classified, with a wide variety of sleep duration, midawake counts, and midawake duration between them (*SI Appendix*, Tables S11 and S12). By focusing on sleep duration, these insomnia clusters could be classified into three groups—the long sleep duration (cluster 1), normal sleep duration (clusters 3a and 4b-5), and short sleep duration (other clusters) groups. Several differences between insomnia with normal sleep duration and with short sleep duration have been reported (50, 51). Interestingly, insomnia with short sleep duration has been associated with impaired neurocognitive functioning (50), while insomnia with normal sleep duration has been associated with an anxious–ruminative profile (51). Those differences demonstrate that it is important to describe subjects with insomnia in more detail based on how much they sleep. Moreover, insomnia clusters with normal or short sleep duration can be subdivided based on midawake counts. Clusters 3a and 3b and clusters 3b-1 and 3b-2, derived from cluster 3b, are characterized by fragmentation of sleep caused by more frequent midawake. Cluster 3a shows the most fragmented sleep among these clusters, but sleep duration was normal. Clusters 3b, 3b-1, and 3b-2 also show fragmented sleep, with clusters 3b-1 and 3b-2 being more fragmented. The number of awakenings is usually measured by PSG but is rarely used in studies on insomnia. Our results suggest that the number of awakenings might distinguish heterogeneous sleep phenotypes that were otherwise considered as the same phenotype (52). The comparison between clusters 3b-1 and 3b-2 demonstrates the variation of duration of each midawake. Cluster 3b-1 has 3.09 times more long-term midawake (longer than or equal to 60 min) but 1.39 times less short-term midawake (less than 60 min) than the average of all data, indicating that subjects in cluster 3b-1 could sleep deeply but could not fall asleep smoothly again once they woke up. On the other hand, cluster 3b-2 has both long-term and short-term midawake, which is why this cluster shows the shortest sleep duration among insomnia-like clusters. The phenotypes of subjects with long-term midawake (e.g., clusters 3b-1 and 3b-2) could be considered as insomnia with difficulty in both maintaining and initiating sleep (53). Both clusters 4b-4 and 4b-5 have normal midawake counts, but what this characteristic means varies between them. Cluster 4b-4 has the most amount of short-term midawake among the insomnia-like clusters. The combination of normal midawake counts and short-term midawake suggests that subjects in cluster 4b-4 wake and fall asleep repeatedly and frequently, indicating that they might not have trouble falling asleep again after midawake but have a problem in maintaining sleep. Cluster 4b-5 has normal counts of midawake and little short-term midawake, indicating that subjects in this cluster suffer from insomnia-like symptoms less often than those in other insomnia clusters. The *International Classification of Sleep Disorders*, Third Edition (18) and the *Diagnostic and Statistical Manual of Mental Disorders*, Fifth Edition (33) define insomnia as a sleep phenotype with sleep difficulty on more than three nights per week, suggesting that the subjects in cluster 4b-5 do not fully meet this criterion and could be classified as preinsomnia. In the context of insomnia, individual records for a longer period would also be useful. Taken together, seven clusters show various insomnia phenotypes, ranging from well-known to undefined types, suggesting that quantitative, detailed, and accurate sleep analysis using accelerometers will also contribute to the classification as well as diagnosis of diseases.

We propose a pipeline for drawing a landscape of sleep phenotypes using a systematic and unbiased clustering method. By linking it with information of different modalities, such as present illness, past medical history, medications, education, occupations, lifestyle habits (e.g., alcohol intakes, smoking, diet), blood biochemistry, and genomics, the human sleep landscape could become more comprehensive and accurate. Some of this information can be obtained from the UK Biobank. The UK Biobank contains a series of diagnostic data classified following the International Classification of Diseases, 10th revision categories. Thus, it would be a promising next step of this study to analyze the relationship between the subjects in each cluster and their medical history, although the dataset in the UK Biobank is not inclusive for all subjects. For example, for subjects with a medical history of sleep apnea syndrome or other sleep-related disorders, it would be interesting to use this information for an annotation of clusters, which will expand the current human sleep landscape.

We also note that accurate and long-term sleep measurement could help us to diagnose psychiatric disorders complicated by sleep disorders because detailed sleep phenotypes of such disorders vary according to the underlying psychiatric condition (54) and may work as digital biomarkers. For example, 15% of depressed patients complain of hypersomnia, and about 70% complain of insomnia with difficulty in initiating or maintaining sleep (55). A study reported that 11 first episode and neuroleptic-naive patients showed prolonged sleep latency but normal sleep duration, indicating that these patients had insomnia with difficulty in initiating sleep (56). Interestingly, some studies reported that patients with schizophrenia show decreased TST and significant disruption in maintaining sleep (57). As psychiatric disorders, including schizophrenia, are understood as a spectrum, it may be possible to segregate psychiatric disorders at a higher resolution by classifying them based on the symptoms of complicating sleep disorders. Moreover, medication strategies depending on complicating sleep disorders may lead to better pharmacologic treatments of psychiatric disorders. In this context, utilizing the rich dataset stored in the UK Biobank including the diagnostic results of mental disease, will enable a pilot study for investigating the relationship between the human sleep landscape and psychiatric disorders.

## Materials and Methods

### Data Acquisition.

Thirty-six simultaneously measured PSG and Axivity data were obtained from healthy human volunteers at the monitoring facility in the University of Tokyo and used to train and validate the sleep/wake classification algorithm. To evaluate the accuracy of the nonwear detection algorithm, we collected Axivity data of 20 subjects (267 d of data in total). Wearing and nonwearing periods were judged using the time stamp records when the subjects were not wearing the device.

### The UK Biobank Dataset.

Axivity data (index [ID]: 90001) recording along with information regarding sex (index [ID]: 31), year of birth (ID: 34), and month of birth (ID: 52) were downloaded from the UK Biobank. Age was calculated by taking the difference between the first day of the subject’s birth month and the first day of acceleration data.

## Supplementary Material

Supplementary File

## Data Availability

Acceleration data used for the large-scale analysis in this study were previously deposited in the UK Biobank (project ID: 48357).
