# Supplementary material for: The 103,200-arm acceleration dataset in the UK Biobank revealed a landscape of human sleep phenotypes
Source: Proc Natl Acad Sci U S A. 2022 Mar 18;119(12):e2116729119. doi: 10.1073/pnas.2116729119 (PMC8944865; doi:10.1073/pnas.2116729119)
Supplement: Supplementary File [file pnas.2116729119.sapp01.pdf]

www.pnas.org

Supplementary Information for

The 103,200-arm acceleration dataset in the UK Biobank revealed a landscape of human sleep phenotypes

Machiko Katori, Shoi Shi, Koji L. Ode, Yasuhiro Tomita and Hiroki R. Ueda

Hiroki R. Ueda
E-mail: uedah-tky@umin.ac.jp

This PDF file includes:

Supplementary text
Figs. S1 to S12 (not allowed for Brief Reports)
Tables S1 to S12 (not allowed for Brief Reports)
SI References

Supporting Information Text

Materials and Methods

Data acquisition. In this study, PSG data and Axivity (AX3, a wristband-type triaxial accelerometer, Axivity Ltd., Hoults Yard, UK) triaxial acceleration data were obtained in the University of Tokyo. Thirty-six PSG data and triaxial accelerometer data of one-night measurement were used to train and validate the sleep/wake classification algorithm. To evaluate the accuracy of nonwear detection algorithm, we collected Axivity data of 20 subjects (267 days data in total). Wearing and nonwearing periods were judged using the timestamp records when the subjects were not wearing the device.

Participants (cumulative counts) who reported no present and past diagnosis of any sleep disorders, mental illnesses, and neurodegenerative diseases were recruited. No participants declared taking medicines that may affect sleep, such as cold medicine. More than two measurements per participant were allowed. The study protocols were approved by the Research Ethics Committee, Graduate School of Medicine, the University of Tokyo (No. 2018174NI and No. 11281). Informed consent was obtained from every subject for each measurement. More information about the subjects has been provided in Table S1, Table S2, and Table S3.

PSG data were recorded using a wireless portable PSG system (SOMNOscreen plus, SOMNOmedics GmbH, Germany) at the monitoring facility in the University of Tokyo. Sleep staging for every 30 seconds with PSG records was conducted manually by trained experts (Fukuda Denshi Co. Ltd, Japan). The sampling rate and dynamic range of Axivity were set to 50 or 100 Hz and ± 8 g at 10-bit resolution, respectively.

Sleep/wake classification algorithm. The sleep/wake classification algorithm used in this study is based on a machine learning-based algorithm created for a triaxial accelerometer (ACCEL) (1). To validate and optimize the parameters of original algorithm to the Axivity data, 27 PSG and Axivity data were used. The original algorithm uses the power spectrum of jerk as input data (Fig. S1*A*) and XGBoost with six hyperparameters: *learning_rate*, *gamma*, *colsample_bytree*, *subsample*, *max_depth*, and *min_child_weight*. These parameters were optimized by using Bayesian optimization (Fig. S1*D*). Each parameter was allowed to have values within the range, [0, 1], [0, 5], [0.01, 1], [0.01, 1], [1, 30], and [1, 30], respectively. The parameter set of six hyperparameters was evaluated by leave-one-out cross-validation, where 26 data were used for training XGBoost with a parameter set, and 1 data was used for validation. The performance of algorithm was evaluated based on the summation of accuracy and F measure. We used Bayesian optimization and iterated 2,000 times to tune the algorithm. F measure was calculated as $2 \times precision \times recall/(precision + recall)$, where wake was calculated as true and sleep as false. The optimal parameters were as follows: $learning\_rate = 0.07$, $gamma = 4.88$, $colsample\_bytree = 0.86$, $subsample = 0.93$, $max\_depth = 23$, and $min\_child\_weight = 5$. The program for Bayesian optimization was adopted from https://github.com/fmfn/BayesianOptimization.

The performance of sleep/wake classification algorithm was calculated by leave-one-out cross-validation. The predicted sleep/wake time series data were compared to PSG-based sleep/wake time series data (ground truth) epoch to epoch, and the average scores are 93.24% (accuracy), 85.69% (F measure), 97.20% (sensitivity), 82.19% (specificity), and 81.03% (Cohen's kappa). Sensitivity and specificity represent the performance in terms of sleep and wake detection, respectively. The performance of sleep/wake classification algorithm was also evaluated for WASO and TST. We calculated TST in predicted sleep/wake time series data by calculating the total sleep time between sleep onset and sleep offset, where the first sleep epoch that sustained more than 15 minutes was defined as the sleep onset and the last sleep epoch as sleep offset. The performance of TST/WASO calculation under varying thresholds of sleep onset from 0 to 30 minutes with 2.5-minute increments is shown in Fig. S2*A*-*D*. WASO was calculated as the length of wake duration between sleep onset and sleep offset.

Nonwear detection algorithm. Nonwearing period was predicted based on the standard deviation and the value range of each accelerometer axis calculated for every 60-minute block shifted by 15 minutes (2). This algorithm detects a block as a nonwearing period if the standard deviation is less than the threshold in at least two axes or if the range is less than the threshold in at least two axes. Twenty triaxial accelerometer data (267 days in total) were acquired for validation of nonwear detection (Table S2). For these measurements, the participants were asked to keep the records of the time when they were not wearing the device, which was used as the ground truth. The standard deviation and range of each axis were calculated for every 60-minute period. The process to make blocks for determining thresholds was referred to that in the previous study (2). The threshold values proposed in the previous study (2) were adapted in this study (Fig. S1*E, F*).

Verification of algorithms and thresholds for UK Biobank analysis. In order to evaluate the effect of difference in sampling frequency of Axivity on the performance of sleep/wake classification, triaxial acceleration data sampled at 100 Hz and PSG data were obtained ($n = 12$, Table S3). To compare the performances, the 100 Hz triaxial acceleration data were converted to 50 Hz before being input into the sleep/wake classification algorithm trained by 50 Hz data (Fig. S1*H*). The method converting 100 Hz data to 50 Hz data was used in the analyses of the UK Biobank dataset (Fig. S1*H*).

To evaluate the performance of sleep episode detection, epochs were randomly sampled from the triaxial acceleration data and connected to create a pseudo sleep/wake time series data (Fig. S3*A*). To eliminate bias due to epochal selection, 1,000 validations were performed on each of the two data groups. Pseudo sleep/wake time series data containing 10 hours of sleep or wake were created, and the pseudo wake or sleep data were inserted in between, respectively. The length of the inserted data was varied from 0 to 100 minutes at a 1-minute step. Sensitivity when sleep pulse pseudo data were classified by the sleep/wake classification algorithm is shown in Fig. S3*B*. Specificity when wake pulse pseudo data were classified is shown in

Fig. S3*C*. Both scores reach 90% the first time when the threshold is 10 minutes when the threshold is changed from 0 minute to 100 minutes by a 5-minute step. These results demonstrate that epochs over 10 minutes, which are continuously classified as sleep or wake, are sufficiently reliable as characteristics of sleep patterns.

UK Biobank dataset. Axivity triaxial acceleration data (ID: 90001) recording as up to seven-day continuous measurements, along with information regarding sex (ID: 31), year of birth (ID: 34), and month of birth (ID: 52), were downloaded from the UK Biobank. Age was calculated by taking the difference between the first day of the subject's birth month and the first day of acceleration data. In this analysis, one day was defined as the sleep/wake time series data from a noon to the next noon and named noon-to-noon data. Individual records from 103,200 subjects were analyzed in this study.

The triaxial acceleration data were converted into sleep/wake time series data as follows. The nonwear detection algorithm was applied to the all dataset. The noon-to-noon data containing less than five hours of nonwearing period were considered as qualified noon-to-noon data. This criterion was decided by referencing previous studies (3–5). The maximum counts of continuous qualified noon-to-noon data ranged from zero to six as shown in Fig. S4*A*, and individual records with two continuous noon-to-noon data showed the lowest ratio. We selected subjects with more than three continuous qualified noon-to-noon data and used their records (91,765 individual records) for the analysis of sleep index (Fig. 3*A*).

Extraction of common sleep indexes. Due to the low sensitivity and specificity of determining wake episodes and sleep episodes shorter than 10 minutes (see also **Verification of algorithms and thresholds for UK Biobank analysis**), sleep episodes shorter than 10 minutes were converted to wake episodes and vice versa. If the length of the time gaps between sleep periods was less than 60 minutes, we connected them as a sleep window (4). According to the length of each sleep window, we named them as a short sleep window or a long sleep window. The threshold between the short and long sleep windows was determined by plotting the distribution of length of sleep window and fitting the distribution with the exponential curve and the Gaussian curve (Fig. S4*B*). The intersection point of two curves, 3.75 hours, was determined as the threshold. Then, non-qualified noon-to-noon data were removed and 21 sleep indexes were calculated for qualified noon-to-noon data (522,826 days). ST and WT within each long or short sleep window were measured as ST long mean, WT long mean, ST short mean, and WT short mean in each qualified noon-to-noon data. Each type of value between multiple noon-to-noon data was summarized as the mean (MN) and the standard deviation (SD) within each individual record. Each window was considered to belong to the day with the middle of the window to avoid double counts. Finally, sleep percentage was determined as the percentage of sleep amount during each individual record.

Extraction of rhythm-related sleep indexes. ***Period***: The chi-square periodogram (6) was applied to the sleep/wake time series data of qualified noon-to-noon data in the range 5 to 35 hours with a 0.1-hour step. The significance level of chi-square periodogram was determined as 0.01, as on the reference studies (6, 7). The maximum significant point was determined as period of sleep/wake oscillation (Fig. 2*D*). If there was no significant peak, period was set to zero. ***Amplitude***: Sleep/wake time series data were divided into 10-minute blocks. The wake duration was calculated in every block without nonwearing periods. The standard deviation and mean of the wake duration between blocks were calculated, and a coefficient of variation (standard deviation/mean) was defined as amplitude as previously described (8) (Fig. 2*E*). ***Phase***: Kronauer's model based on the van der Pol limit cycle was used to extract sleep-wake cycles phases. Sleep/wake time series data were converted to binary data (sleep as 0 and wake as 1), where the nonwearing period was considered as missing value. In order to fit the limit cycle curve to the binary data, the limit cycle oscillating at 0∼1 was shifted by 30 seconds, and the mean square error from the binary data was calculated to determine the best-fitted curve. The duration between the minimum point of the best-fitted curve and the last noon was determined as phase of each day. The fitting of phase is calculated for a 24-hour period, regardless of the value of period estimated in sleep-index analysis. Phase MN and SD were calculated to summarize phase features (Fig. 2*F*).

Finding the function form to fit each sleep index. To find the function form that fits the distribution of each sleep index, we tested all functions implemented in scipy.stats to the distribution of sleep indexes except for the number-related sleep indexes (long sleep window # and short sleep window #) and SD features (Fig. 3*B-G*, Fig. S4*G, I, M, R*). The ten curves, "rv_continuous", "rv_histogram", "levy_stable", "ncx2", "crystalball", "geninvgauss", "vonmises", "gausshyper", "chi", and "powerlaw", were excluded due to errors during calculation. Each curve was fitted using maximum likelihood estimation for each sleep index, and the curve with the lowest Akaike information criterion score was selected as the best-fitted curve for each sleep index. Details about the fitting curves are available at https://docs.scipy.org/doc/scipy/reference/stats.html.

Clustering process for whole dataset and outlier dataset. The clustering processes consisted of three steps: z score normalization, dimension reduction, and clustering (Fig. 4*A*). Z score normalization was performed on each sleep index to normalize the mean and standard deviation of each sleep index to zero and one, respectively. We tested four dimension reduction methods: PCA, t-SNE, UMAP (9), and a combination of PCA and UMAP (Fig. 4*B, C*, Fig. S5*A-D*). In the first three approaches, the 21-dimensional z score data were compressed to three-dimensional data. In the last approach, the 21-dimensional z score data were converted to 21 principal components (PCs) by PCA, and then n PCs were further compressed to three-dimensional data by UMAP (Fig. S5*D*). DBSCAN was used as the clustering method.

To perform hierarchical clustering, the individual records within a cluster were applied to the clustering process. Z score normalization was conducted in every clustering process to normalize the target individual records. The repetition of this clustering process continued as long as at least two of the newly generated clusters had a size equaling 20 or larger.

The mean of each sleep index was calculated in each identified cluster (Fig. S6*B*). The mean was regarded as the characteristic of each cluster. We divided clusters obtained in hierarchical clustering into five groups depending on Clusters 1 to 5 generated by the first clustering process. Ward's method was used to measure the distances among clusters within their mother cluster (i.e. Clusters 1 to 5), where the weight was determined by the each cluster size (Fig. 5*A*). The distribution of distances among clusters was represented as a histogram ranging from the minimum distance to maximum distance. The bin size was determined by increasing the number of bins until the gap point, a bin with zero value, occurred. The clusters were merged if their distances were lower than the median value of gap (Fig. 5*A*).

An outlier dataset was selected by considering six sleep indexes: ST long MN, WT long MN, ST short MN, phase MN, period, and amplitude (Fig. 6*A*). Individual records with abnormal values (upper or lower 2.28 percentiles) in at least one of six sleep indexes were selected as an outlier dataset from Clusters 3b and 4b (Fig. 6*B*) or all clusters (Fig. S12*E, F*).

Hyperparameter setting in clustering. Default parameters in the UMAP package were used for all analyses. This package is available at https://umap-learn.readthedocs.io/en/latest/index.html. Among the hyperparameters, *n_neighbors*, (default: 15) and *min_dist* (default: 0.1) manage structures in a high- or low-dimensional field. We tested different values of those two parameters with the whole data (Fig. S6*A*).

The DBSCAN package implemented in sklearn was used with the default values in all parameters except for *min_samples*, which is the parameter to form a dense region (10, 11). The default value, 5, was used in the whole clustering, and 99 or 115 were used in the outlier clustering of Clusters 3b and 4b or of all clusters.

Representative plot. Two types of representative plot were shown in this study. The first type focused on one sleep index. Individual record with abnormal values (upper or lower 2.28 percentiles) in the focused sleep index were selected as candidates. Euclidean distances from the mean of the whole dataset were calculated using 21 sleep indexes, and the individual record that was closest to the mean was shown as the representative plot (Fig. 3*B-G*). The other type was the representative plot in each cluster. Distances from the mean of cluster data were calculated, and representative plots were displayed in ascending order of distance (Fig. 5*E, F, H, I, K*, Fig. 6*I-P*, Fig. S8, Fig. S11).

Analysis of age and sex information. The sex percentage was calculated for each cluster, the whole dataset, and the outlier dataset from Clusters 3b and 4b. Age was categorized into four groups, the 40s, 50s, 60s, and 70s.

References

1. KL Ode, et al., A jerk-based algorithm ACCEL for the accurate classification of sleep–wake states from arm acceleration. *iScience* **25**, 103727 (2022).
2. VT van Hees, et al., Separating Movement and Gravity Components in an Acceleration Signal and Implications for the Assessment of Human Daily Physical Activity. *PLoS One* **8**, e61691 (2013).
3. HS Dashti, et al., Genome-wide association study identifies genetic loci for self-reported habitual sleep duration supported by accelerometer-derived estimates. *Nat. Commun.* **10**, 1100 (2019).
4. VT van Hees, et al., Estimating sleep parameters using an accelerometer without sleep diary. *Sci. Rep.* **8**, 12975 (2018).
5. A Doherty, et al., Large Scale Population Assessment of Physical Activity Using Wrist Worn Accelerometers: The UK Biobank Study. *PLoS One* **12**, e0169649 (2017).
6. PG Sokolove, WN Bushell, The chi square periodogram: Its utility for analysis of circadian rhythms. *J. Theor. Biol.* **72**, 131–160 (1978).
7. YL Huang, et al., Age-associated difference in circadian sleep–wake and rest–activity rhythms. *Physiol. Behav.* **76**, 597–603 (2002).
8. GA Sunagawa, et al., Mammalian Reverse Genetics without Crossing Reveals Nr3a as a Short-Sleeper Gene. *Cell Rep.* **14**, 662–677 (2016).
9. L McInnes, J Healy, J Melville, UMAP: Uniform Manifold Approximation and Projection for Dimension Reduction. *arXiv* (2018).
10. E Martin, K Hans-Peter, S Jörg, X Xiaowei, A Density-Based Algorithm for Discovering Clusters in Large Spatial Databases with Noise. *Kdd* **96**, 226–231 (1996).
11. E Schubert, J Sander, M Ester, HP Kriegel, X Xu, DBSCAN Revisited, Revisited: Why and How You Should (Still) Use DBSCAN. *ACM Trans. Database Syst.* **42**, 1–21 (2017).

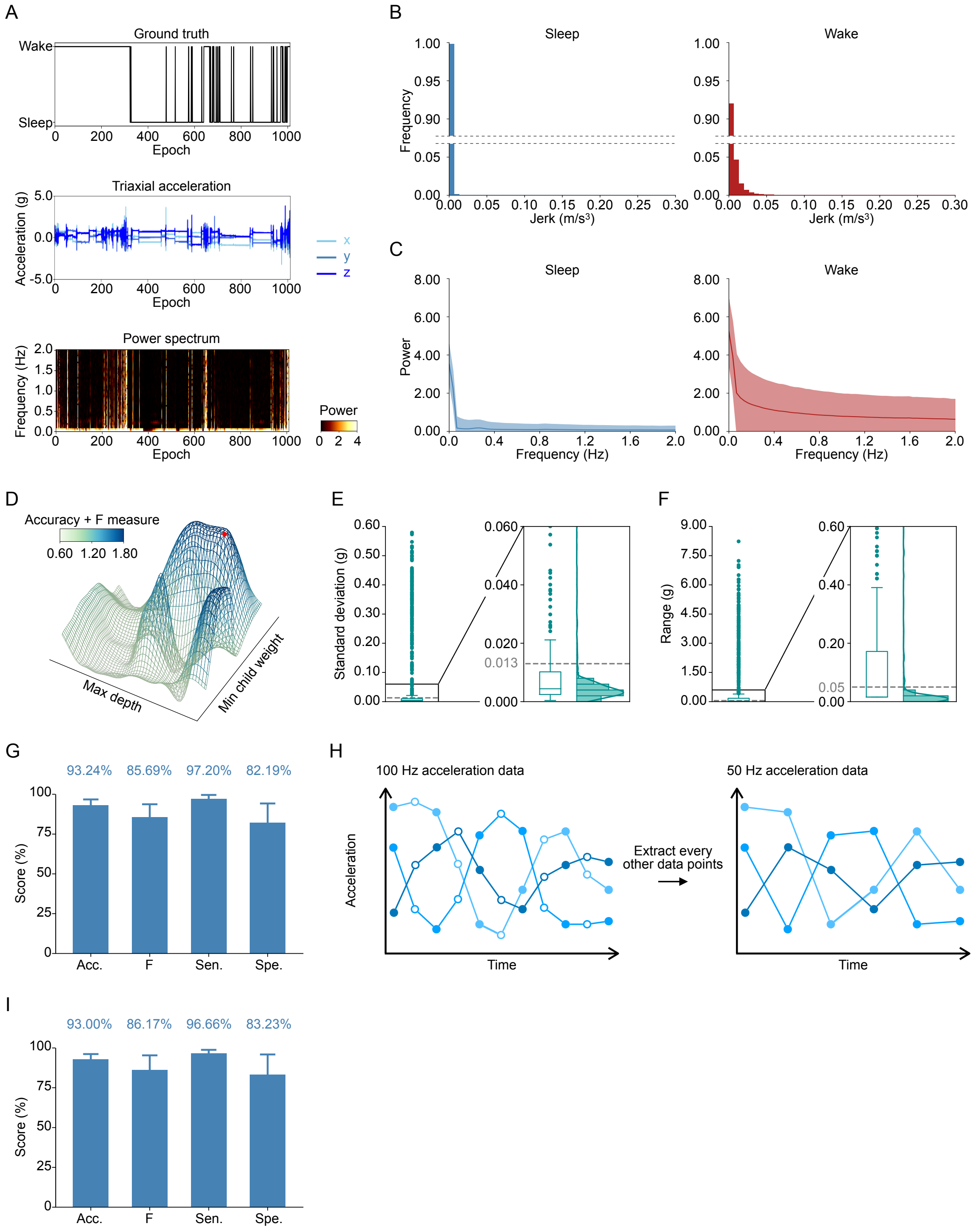

Fig. S1. Validation of sleep/wake classification algorithm. (*A)* An example of ground truth, triaxial acceleration, and power spectrum. In the middle panel, three axes of raw acceleration data are plotted in different colors. The power spectrum in the bottom panel is shown by heatmap, where the lighter color shows a higher value. (*B)* The histogram of jerk among sleep and wake epochs. (*C)* The power spectrum among sleep and wake epochs. The solid line and the shadowed area shows the mean and the standard deviation. (*D)* Results of hyperparameter tuning of XGBoost used in the sleep/wake classification algorithm as 3D plot of summation of accuracy and F measure with two hyperparameters as x and y axes. The max depth and min child weight represent the maximum tree depth for base learners and the minimum summation of instance weight (hessian) needed in a child, respectively. The red circle shows the point of maximum score, whose parameter values were used for the sleep/wake classification algorithm. (*E, F)* The histogram and distribution of standard deviation and range in nonwearing periods. The gray dashed lines show the threshold used in this study. (*G)* The performance of sleep/wake classification algorithm. (*H)* Flow of thinning process for converting 100 Hz to 50 Hz acceleration data. The left panel shows acceleration data sampled by 100 Hz, where each dot shows data point. Data points plotted as white dots are excluded, then the 100 Hz acceleration data is converted to 50 Hz acceleration data shown in the right panel. (*I)* The result when the sleep/wake classification algorithm trained by 50 Hz acceleration data were applied to reprocessed 100 Hz acceleration data. The 100 Hz acceleration data were converted to 50 Hz acceleration data by the thinning process shown in (*H)* as the preprocessing, and then the algorithm was applied to the converted acceleration data. Acc: Accuracy. F: F measure. Sen: Sensitivity. Spe: Specificity.

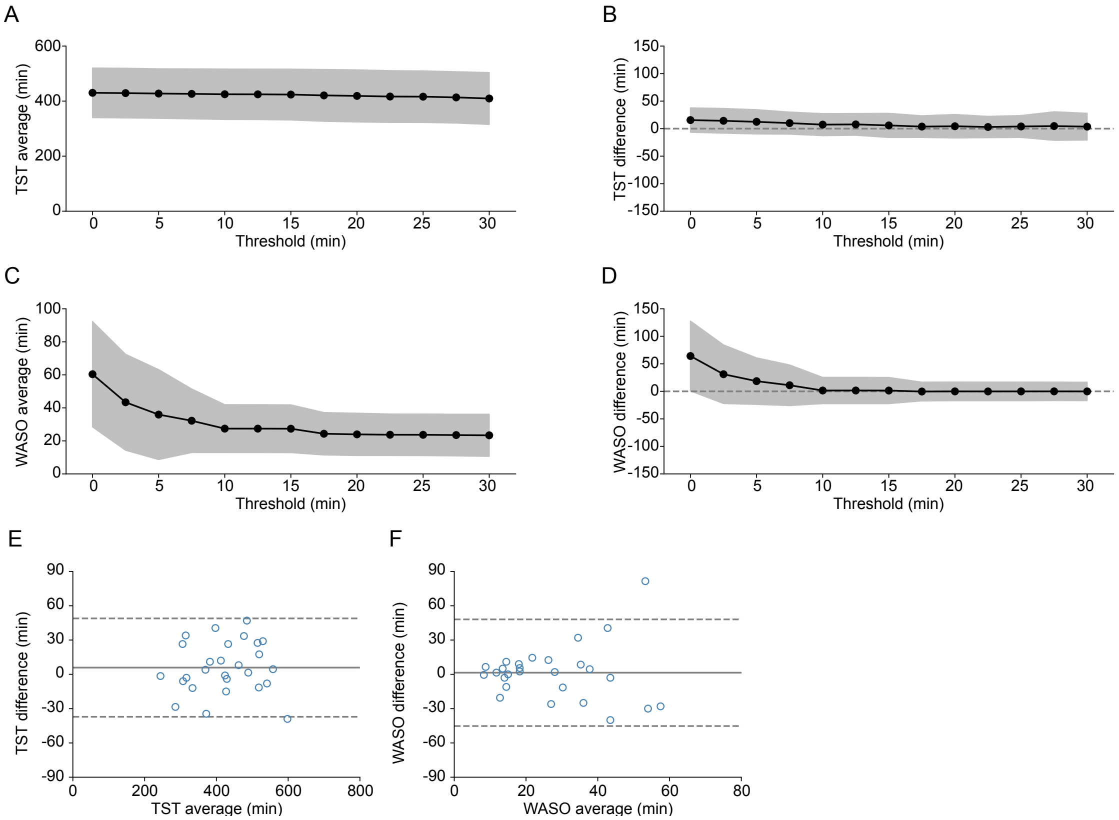

Fig. S2. Evaluation of performance of sleep/wake classification algorithm by TST and WASO. (*A-D)* The results of TST and WASO under varying threshold used to detect sleep onset. The threshold was changed from 0 to 30 minutes by a 2.5-minute step. The black dot and solid line shows the mean, and the shaded area shows the standard deviation among subjects. (*E, F)* Bland-Altman plots of TST and WASO. The solid gray lines show the mean, and the dashed lines represent the mean $\pm$ 1.95 standard deviation.

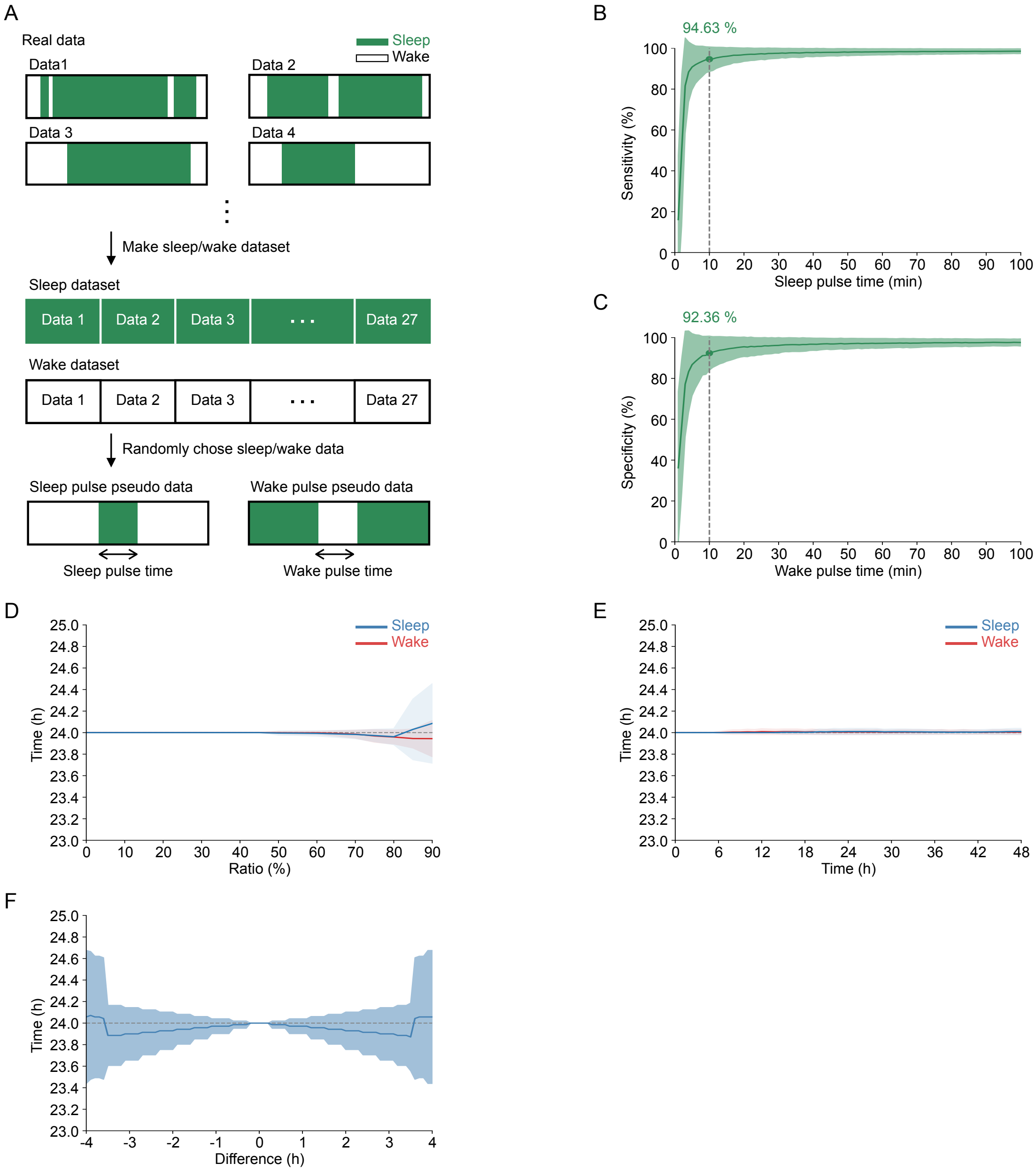

Fig. S3. Validation of sleep indexes extraction. (*A)* Flow diagram to make two types of pseudo sleep/wake time series data, namely, sleep pulse pseudo data and wake pulse pseudo data. The sleep data colored by green and wake data colored by white were grouped as a sleep dataset and wake dataset. Pseudo sleep/wake time series data were made by randomly choosing sleep and wake epochs from the sleep and wake dataset and connecting them. The sleep pulse pseudo data was 10 hours data and consisted of wake epochs except for sleep epochs lasting some time (defined as sleep pulse time) at the middle of data as shown in the left bottom panel. On the opposite, the wake pulse pseudo data consisted of sleep epochs except for wake epochs lasting some time (defined as wake pulse time) as shown in the right bottom panel. (*B, C)* The sensitivity and specificity of sleep/wake classification algorithm with sleep pulse pseudo data and wake pulse pseudo data. The sleep pulse time and wake pulse time were changed from 0 to 100 minutes at a 1-minute step. All conditions were conducted 1,000 times to eliminate bias due to epoch selection. The solid line and the shaded area shows the mean and the standard deviation. (*D, E)* The validation of interpolating nonwearing periods as wake or sleep. The sleep/wake time series data was generated by connected six noon-to-noon data consisted of 11 hours wake followed by eight hours sleep and five hours wake as the standard data. Nonwearing periods were inserted into the standard data in two ways. The first way is randomly choosing epochs and converting them to nonwearing periods. A percentage of nonwearing periods was changed from 0 to 90% by a 5% step. The sleep/wake time series data with random nonwearing periods was generated 100 times in each condition and interpolated to wake or sleep periods, then the chi-square periodogram was applied to these data. Period was about 24 hour with the nonwearing percent lower than 50% in both interpolations to sleep and wake (*D).* The second way is choosing continuous epochs and converting them to nonwearing periods. The duration was changed from 0 to 48 hours by a 1-hour step. The sleep/wake time series data was generated 100 times by changing the start point of nonwearing periods in each condition and interpolated to wake or sleep periods, then the chi-square periodogram was applied to these data. Period was also about 24 hour in both interpolations (*E).* (*F)* The validation of effect of different sleep schedules between workdays and holidays on the chi-square periodogram. To analyze how the different sleep schedules between workdays and holidays affected a period, we set some days as holidays and shifted the 8-hour sleep window. The shift of sleep window was changed from -4 to 4 hours by a 0.1-hour step. In each condition, seven types of holidays were simulated: only the first day, the continuous two days (the first and second, second and third, third and fourth, fourth and fifth, and fifth and last days), and the last day. The simulation sleep/wake time series data with holidays tended to have less than 24-hour period, which was regarded as the effect of difference between workdays and holidays.

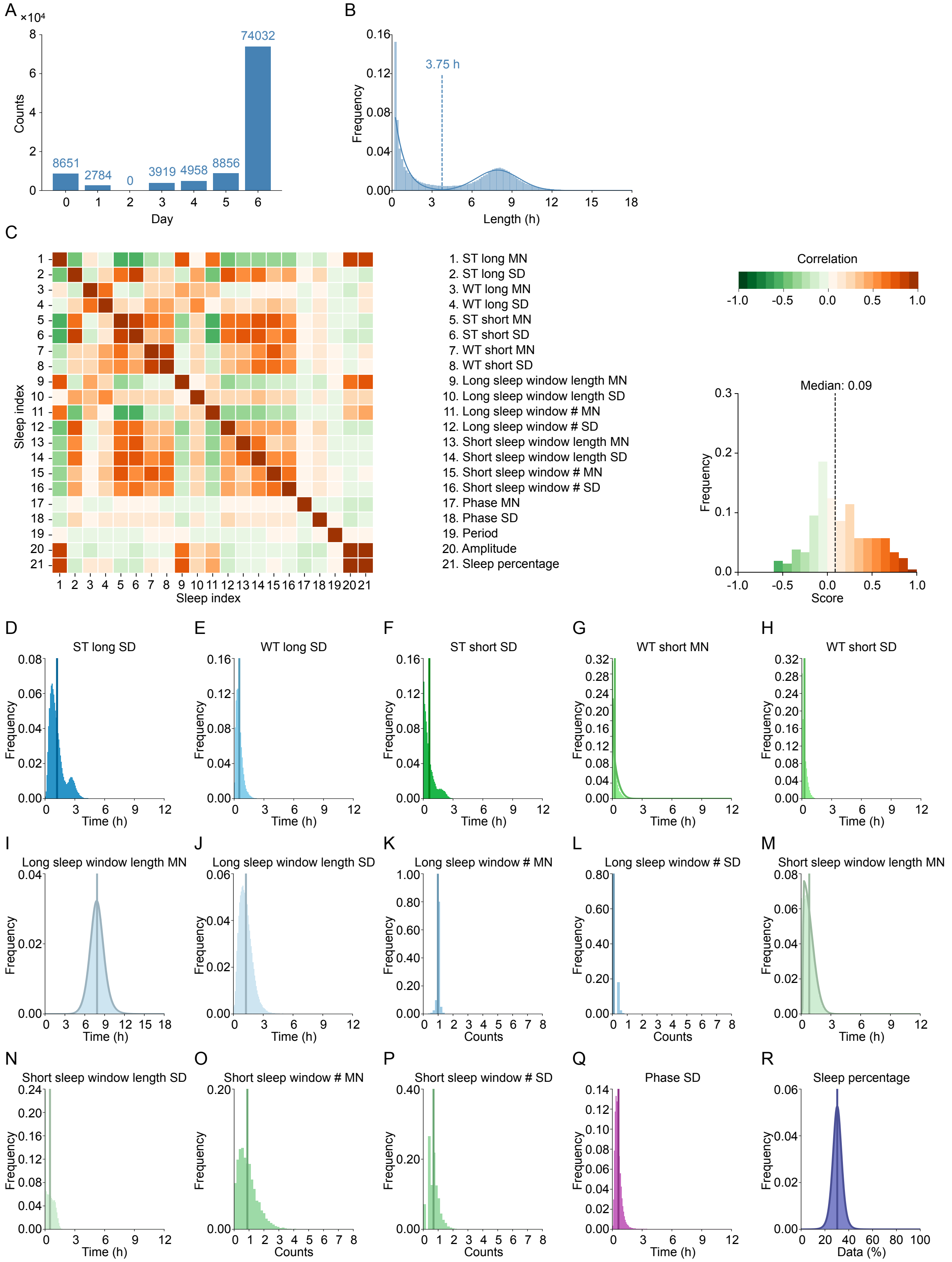

Fig. S4. Results of large-scale sleep analysis. (*A)* Distribution of maximum continuous days. Individual records with more than three continuous days were used for the large-scale sleep analysis. (*B)* Distribution of length of sleep windows. The solid curves shows the exponential curve fitted to the distribution and the Gaussian curve fitted to the distribution with a restriction that the peak of curve and the right ridge were the same. The intersection points of two curves, 3.75 hours, was determined to divide sleep windows into long sleep windows and short sleep windows. (*C)* The left heatmap shows the correlation coefficient between the sleep indexes. The right histogram shows the distribution of correlation coefficient. (*D-R)* Distribution of sleep indexes. The solid line shows the mean. The solid curves shown in (*G*), (*I*), (*M)*, and (*R)* are the curves fitted to each distribution.

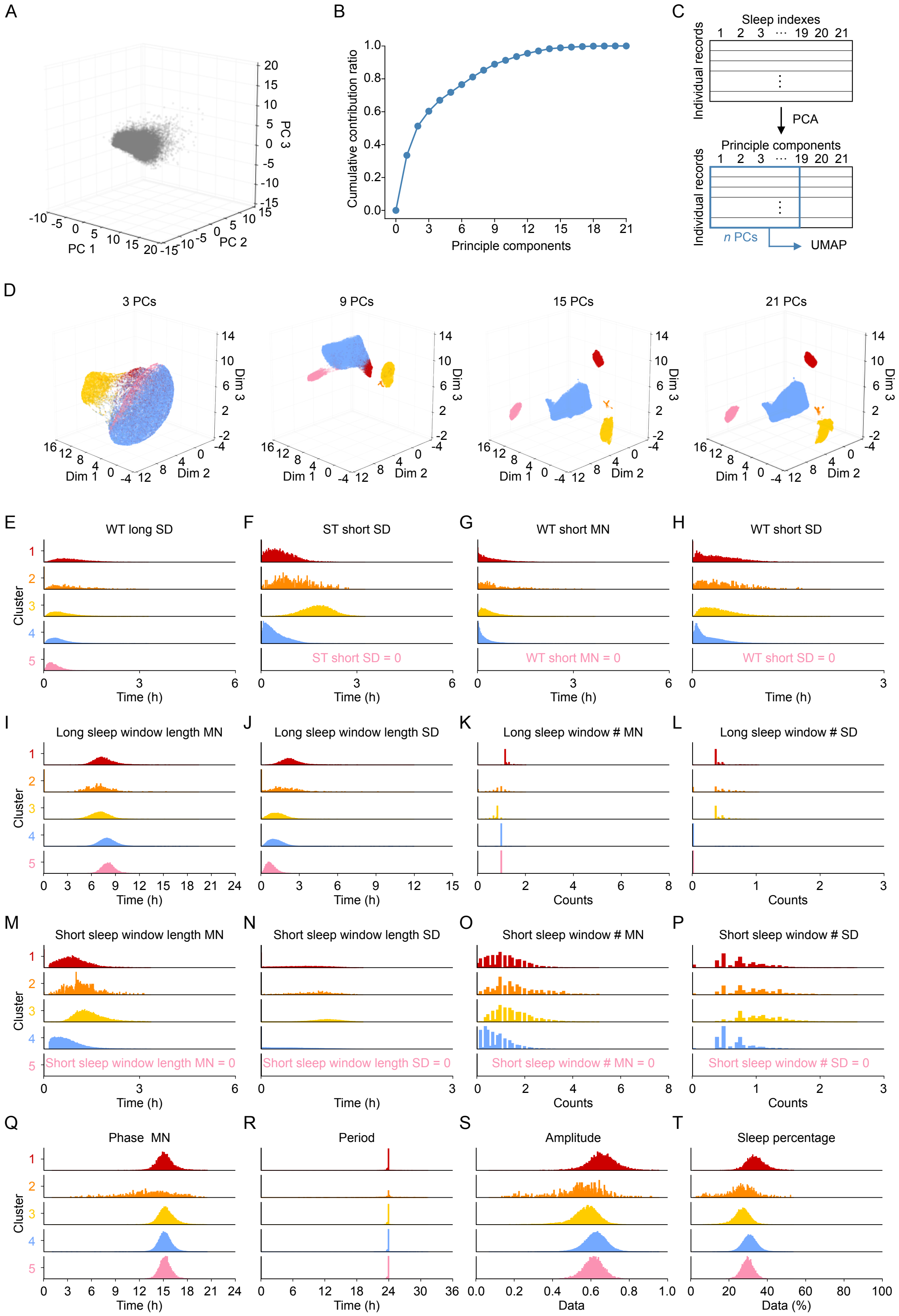

Fig. S5. Results of first clustering. (*A*) The results of PCA of the whole dataset. (*B*) Cumulative contribution ratio of principle components. (*C*) The flow of clustering method combining PCA and UMAP. 21 sleep indexes are converted to 21 principle componenets (PCs) by PCA. *n* PCs are then used to UMAP to express *n*-dimensional data to three-dimensional data. (*D*) The results of clustering, where 3, 9, 15, or 21 PCs were used for UMAP. (*E-T*) Distribution of sleep indexes divided into five clusters. The label of y axis is the cluster name.

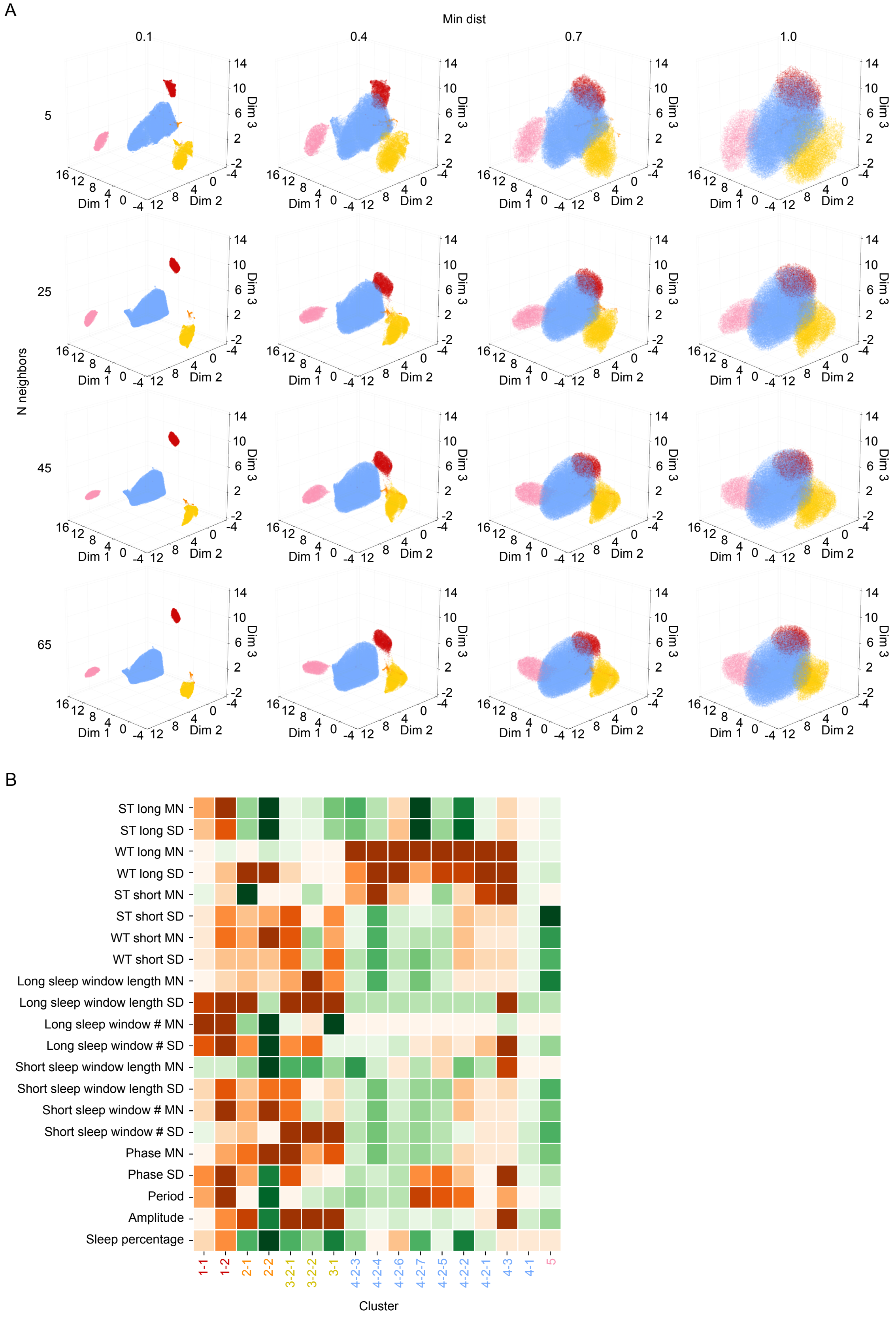

Fig. S6. Effect of hyperparameters and hierarchical clustering result. *(A)* Effect of hyperparameters on the first clustering. Two hyperparameters of UMAP, *n_neighbors* and *min_dist*, were changed. Individual records are plotted as the clusters' colors. *(B)* Heatmap of z score of sleep indexes.

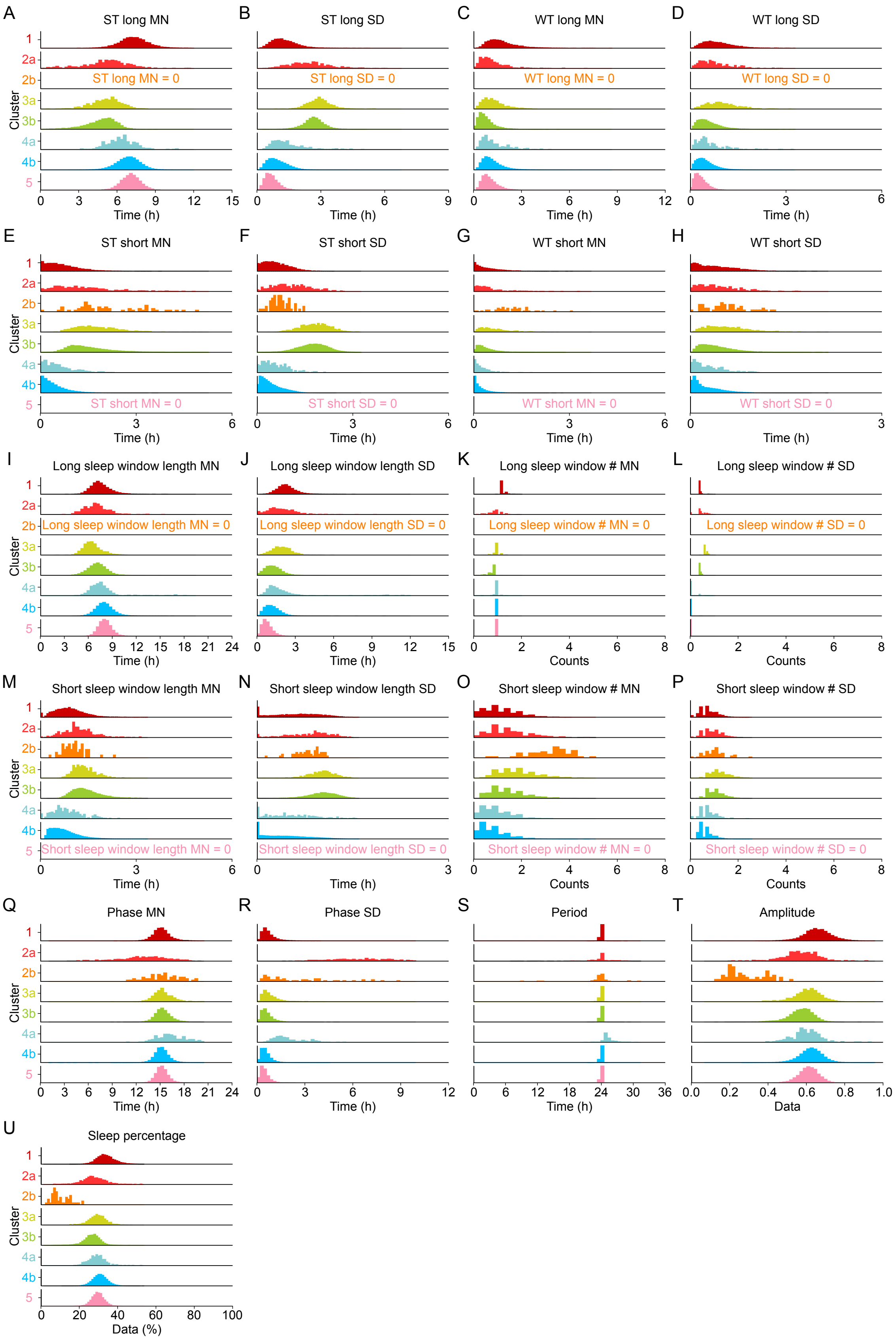

Fig. S7. Distribution of sleep indexes within clusters obtained in the whole clustering. (*A-U*) Distribution of sleep indexes divided into eight. The label of y axis is the cluster name.

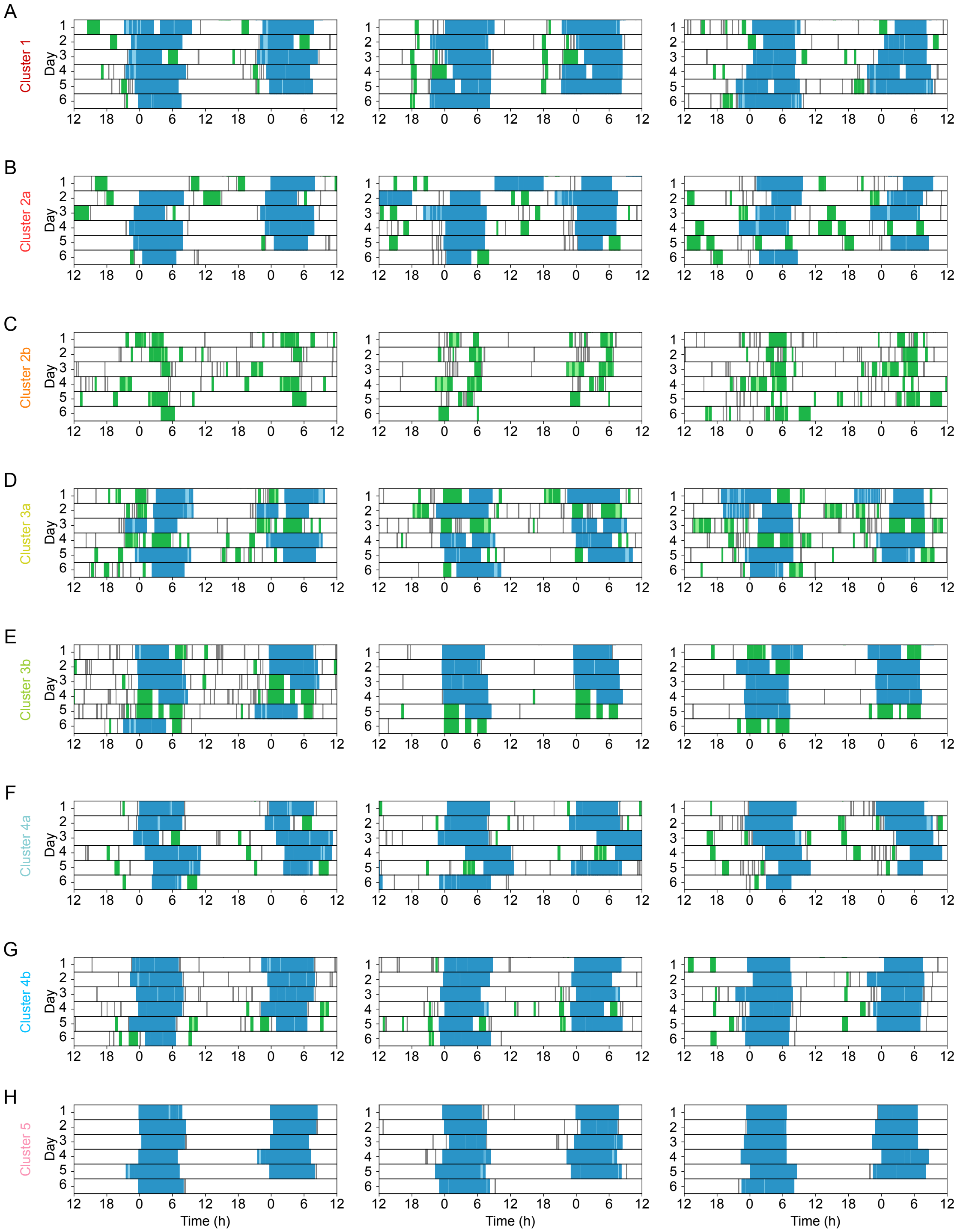

Fig. S8. Representative plots within clusters of the whole clustering. *(A-H)* Representative plots shown as double plot. The label of x axis is time.

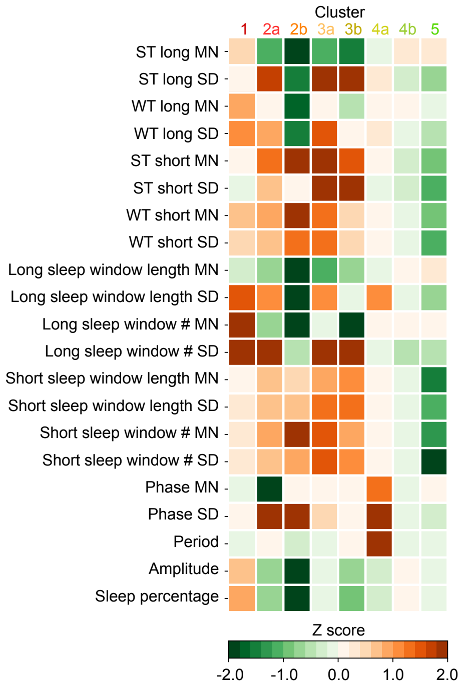

Fig. S9. Heatmap of z score of sleep indexes of each cluster in the whole clustering.

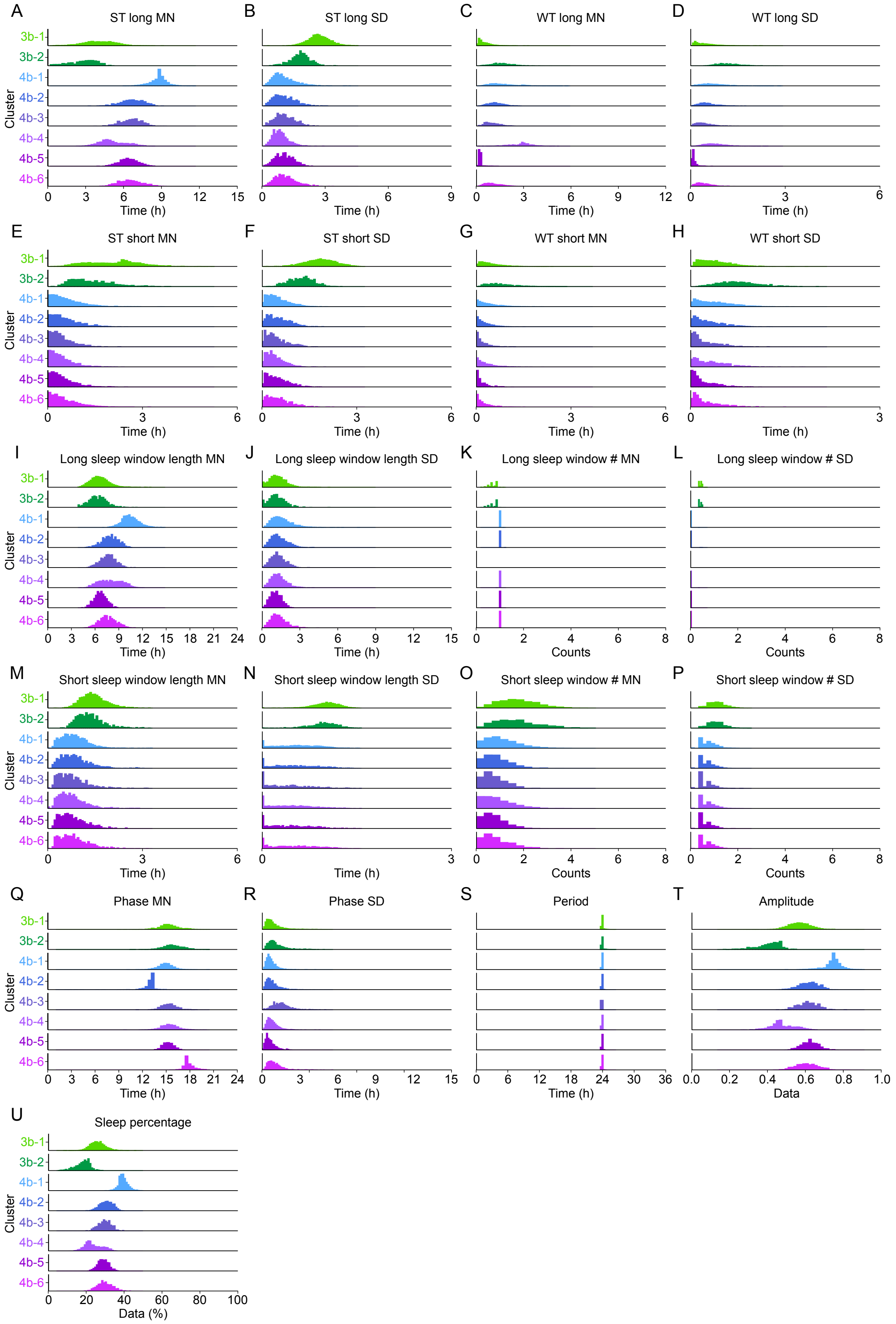

Fig. S10. Distribution of sleep indexes within clusters of the outlier clustering. (*A-U*) Distribution of sleep indexes divided into eight. The label of y axis is the cluster name.

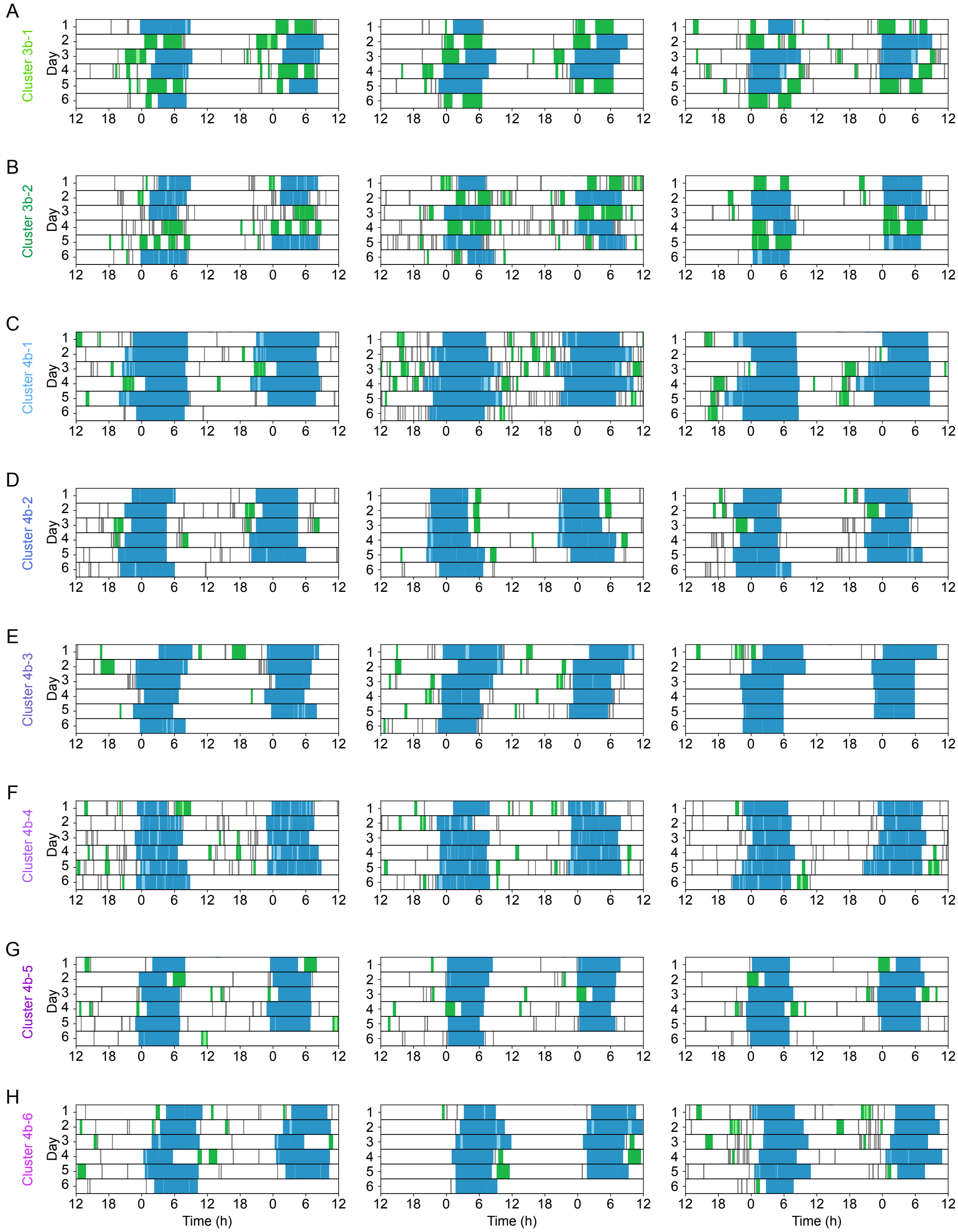

Fig. S11. Representative plots within clusters of the outlier clustering. *(A-H)* Representative plots shown as double plot. The label of x axis is time.

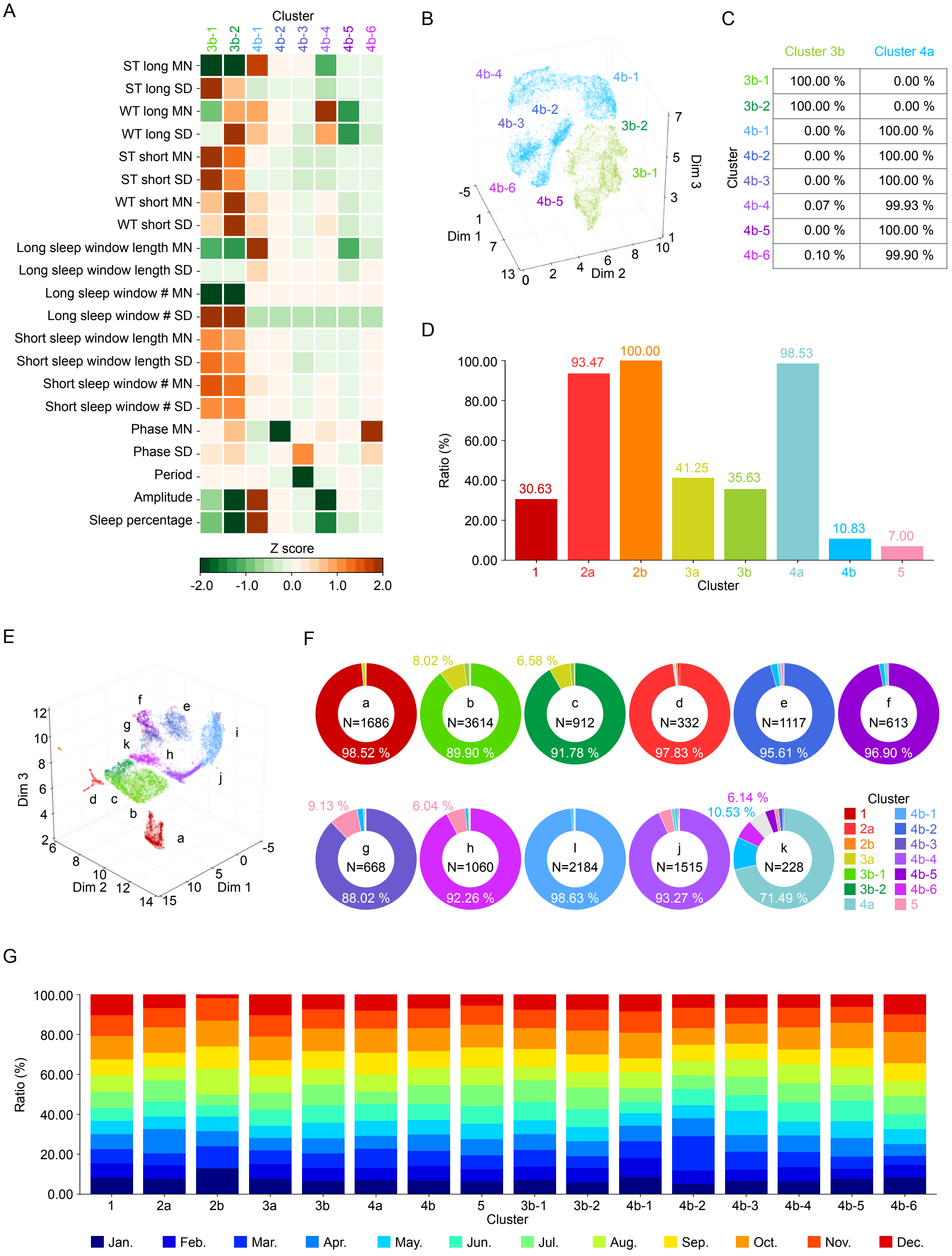

Fig. S12. Supportive data of clusters of outlier clustering. *(A)* Heatmap of z score of sleep indexes. *(B)* The result of outlier clustering, where individual records are colored by the color of whole clustering. *(C)* Ratio of Clusters 3b and 4b contained in each cluster defined in the outlier clustering. *(D)* Ratio of the outlier dataset contained in each cluster defined in the whole clustering. *(E)* The result of clustering using the outlier dataset from all clusters. Individual records plotted by the clusters' colors were divided into 11 clusters named from a to k. *(F)* Ratio of individual records contained in clusters a to k. *(G)* Distribution of month in which the Axivity recording was started.

Table S1. PSG and Axivity recordings (50Hz)

Subject	Sex	Age
1	F	22
2	M	22
3	F	22
4	M	19
5	F	24
6	M	25
7	M	24
8	F	22
9	M	23
10	M	20
11	F	21
12	M	23
13	F	25
14	M	26
15	F	21
16	M	23
17	F	24
18	M	20
19	F	23
20	F	24
21	F	24
22 *	F	23
23 †	F	22
24 ‡	M	21
25	M	23
26	F	24
27	M	22

List of subjects recruited for one-night PSG measurements. All participants wore the Axivity device (50 Hz sampling frequency). The subjects with any symbol (*, †, ‡) also wore Axivity recorded by 100 Hz sampling frequency and the same subject shown in Table S3. F indicates female and M indicates male in all tables.

Table S2. Long-term Axivity recordings for nonwear detection

Subject	Sex	Age	Recording Days
1	M	25	14
2	M	29	14
3	F	23	14
4	F	54	14
5	F	33	14
6	M	30	14
7	M	23	14
8	M	22	14
9	M	21	14
10	F	23	9
11	M	27	14
12	F	23	14
13	F	23	14
14	M	21	14
15	M	38	14
16	M	28	14
17	F	22	14
18	F	24	14
19	F	31	7
20	M	27	14

List of subjects recruited for long-term Axivity recording to validate the nonwear detection algorithm. All participants were asked to record timestamps when they wore or took off Axivity.

Table S3. PSG and Axivity recordings (100Hz)

Subject	Sex	Age
1 *	F	23
2 †	F	22
3 ‡	M	21
4	M	27
5	F	23
6	F	21
7	M	20
8	M	24
9	M	21
10	F	21
11	F	21
12	M	21

List of subjects recruited for one-night PSG measurements. All participants wore the Axivity device (100 Hz sampling frequency). The subjects with any symbol (*, †, ‡) also wore Axivity recorded by 50 Hz sampling frequency and the same subject shown in Table S1.

Table S4. Sleep indexes used in this paper

Sleep index	Name	Feature type
ST long MN	Sleep time long mean	Daily, Common Index
ST long SD	Sleep time long SD	Daily, Common Index
WT long MN	Wake time long mean	Daily, Common Index
WT long SD	Wake time long SD	Daily, Common Index
ST short MN	Sleep time short mean	Daily, Common Index
ST short SD	Sleep time short SD	Daily, Common Index
WT short MN	Wake time short mean	Daily, Common Index
WT short SD	Wake time short SD	Daily, Common Index
Long sleep window length MN	Long sleep window length mean	Daily, Common Index
Long sleep window length SD	Long sleep window length SD	Daily, Common Index
Long sleep window # MN	Long sleep window number mean	Daily, Common Index
Long sleep window # SD	Long sleep window number SD	Daily, Common Index
Short sleep window length MN	Short sleep window length mean	Daily, Common Index
Short sleep window length SD	Short sleep window length SD	Daily, Common Index
Short sleep window # MN	Short sleep window number mean	Daily, Common Index
Short sleep window # SD	Short sleep window number SD	Daily, Common Index
Sleep percentage	Sleep percentage	General, Common Index
Phase MN	Phase mean	Daily, Rhythm-related index
Phase SD	Phase SD	Daily, Rhythm-related index
Period	Period	General, Rhythm-related index
Amplitude	Amplitude	General, Rhythm-related index

Table S5. Means of sleep indexes among the 17 clusters generated by divisive hierarchical clustering

Sleep index / Cluster	1-1	1-2	2-1	2-2	3-1	3-2-1	3-2-2	4-1
ST long MN (h)	7.32	7.91	5.16	0.00	4.73	5.15	5.76	6.88
ST long SD (h)	1.28	2.06	2.50	0.00	2.65	2.92	2.98	0.95
WT long MN (h)	1.77	2.76	1.20	0.00	0.79	1.26	0.90	1.15
ST long SD (h)	0.87	1.51	0.79	0.00	0.58	1.00	0.63	0.48
ST short MN (h)	0.66	1.16	1.33	2.23	1.53	1.78	1.11	0.42
ST short SD (h)	0.60	0.95	1.09	0.73	1.75	1.83	1.96	0.44
WT short MN (h)	0.42	0.83	0.49	1.18	0.41	0.64	0.15	0.22
ST short SD (h)	0.40	0.67	0.44	0.58	0.42	0.58	0.28	0.25
Long sleep window length MN (h)	7.53	7.42	7.00	0.00	7.13	6.51	6.54	8.03
Long sleep window length SD (h)	2.32	2.99	1.94	0.00	1.26	1.94	2.13	1.20
Long sleep window # MN	1.21	1.45	0.91	0.00	0.77	0.98	1.02	1.00
Long sleep window # SD	0.39	0.77	0.46	0.00	0.40	0.63	0.61	0.00
Short sleep window length MN (h)	0.91	1.15	1.21	1.05	1.45	1.38	2.90	0.76
Short sleep window length SD (h)	0.60	0.83	0.82	0.80	1.01	1.02	0.31	0.43
Short sleep window # MN	1.14	1.70	1.50	3.29	1.45	1.84	0.44	0.82
Short sleep window # SD	0.77	1.06	0.95	1.03	1.07	1.23	0.76	0.66
Phase MN (h)	15.20	15.61	12.92	15.34	15.37	15.38	14.80	15.20
Phase SD (h)	0.72	1.03	6.46	2.63	0.67	0.86	0.63	0.56
Period (h)	23.94	23.87	23.99	23.79	23.94	23.92	23.94	23.93
Amplitude	0.66	0.71	0.57	0.28	0.57	0.61	0.61	0.62
Sleep percentage (%)	33.82	38.61	27.57	10.20	26.52	29.50	28.84	30.84

Sleep index / Cluster	4-2-1	4-2-2	4-2-3	4-2-4	4-2-5	4-2-6	4-2-7	4-3	5
ST long MN (h)	6.24	4.85	5.68	6.61	6.45	7.46	5.09	6.97	7.08
ST long SD (h)	1.46	1.07	1.00	1.09	1.00	0.91	1.08	2.75	0.70
WT long MN (h)	1.14	1.95	0.70	0.87	2.04	0.80	2.28	1.71	1.02
ST long SD (h)	0.53	0.75	0.32	0.44	0.92	0.38	0.90	1.20	0.34
ST short MN (h)	0.75	0.82	0.34	0.02	0.26	0.27	0.16	0.79	0.00
ST short SD (h)	0.76	0.60	0.33	0.05	0.32	0.30	0.19	0.76	0.00
WT short MN (h)	0.32	0.45	0.14	0.01	0.09	0.14	0.06	0.34	0.00
ST short SD (h)	0.35	0.42	0.17	0.02	0.11	0.18	0.08	0.37	0.00
Long sleep window length MN (h)	7.38	6.80	6.38	7.48	8.49	8.26	7.36	9.71	8.09
Long sleep window length SD (h)	1.75	1.50	1.13	1.19	1.60	1.10	1.51	5.26	0.80
Long sleep window # MN	1.00	1.00	1.00	1.00	1.00	1.00	1.00	0.95	1.00
Long sleep window # SD	0.00	0.00	0.00	0.00	0.00	0.00	0.00	0.47	0.00
Short sleep window length MN (h)	1.00	0.94	0.63	0.16	0.65	0.67	0.34	1.00	0.00
Short sleep window length SD (h)	0.68	0.77	0.29	0.00	0.30	0.27	0.12	0.68	0.00
Short sleep window # MN	1.09	1.40	0.84	0.13	0.56	0.66	0.56	1.11	0.00
Short sleep window # SD	0.86	0.91	0.67	0.25	0.58	0.60	0.64	0.83	0.00
Phase MN (h)	16.79	15.62	16.08	17.11	14.48	15.98	15.39	17.07	15.22
Phase SD (h)	2.04	1.60	1.24	2.05	1.55	1.69	1.16	2.89	0.48
Period (h)	25.42	25.03	24.77	24.96	25.26	25.17	24.72	25.03	23.93
Amplitude	0.60	0.50	0.56	0.59	0.57	0.66	0.47	0.64	0.61
Sleep percentage (%)	29.62	24.09	25.53	27.89	28.26	32.59	22.30	32.85	29.68

Table S6. Standard deviations of sleep indexes among the 17 clusters generated by divisive hierarchical clustering

Sleep index / Cluster	1-1	1-2	2-1	2-2	3-1	3-2-1	3-2-2	4-1
ST long MN (h)	1.15	1.40	1.84	0.00	1.23	1.16	0.73	0.93
ST long SD (h)	0.57	1.06	1.01	0.00	0.50	0.55	0.35	0.45
WT long MN (h)	0.92	1.18	0.90	0.00	0.51	0.68	0.39	0.60
ST long SD (h)	0.47	0.59	0.52	0.00	0.36	0.48	0.29	0.29
ST short MN (h)	0.52	0.68	0.92	1.18	0.69	0.70	0.35	0.37
ST short SD (h)	0.40	0.54	0.57	0.31	0.47	0.47	0.49	0.33
WT short MN (h)	0.40	0.50	0.47	0.56	0.35	0.46	0.09	0.25
ST short SD (h)	0.31	0.35	0.32	0.32	0.27	0.33	0.16	0.23
Long sleep window length MN (h)	1.22	1.35	1.53	0.00	1.15	1.12	0.61	1.07
Long sleep window length SD (h)	0.74	1.10	1.28	0.00	0.63	0.76	0.59	0.58
Long sleep window # MN	0.09	0.17	0.27	0.00	0.12	0.12	0.09	0.00
Long sleep window # SD	0.04	0.07	0.22	0.00	0.05	0.07	0.06	0.00
Short sleep window length MN (h)	0.48	0.40	0.49	0.35	0.48	0.35	0.42	0.46
Short sleep window length SD (h)	0.38	0.32	0.34	0.20	0.26	0.20	0.22	0.36
Short sleep window # MN	0.70	0.75	0.90	1.02	0.74	0.80	0.13	0.55
Short sleep window # SD	0.35	0.38	0.37	0.44	0.34	0.40	0.18	0.27
Phase MN (h)	1.14	1.69	3.19	2.11	1.06	1.28	0.87	0.89
Phase SD (h)	0.47	0.69	1.90	2.26	0.48	0.61	0.29	0.32
Period (h)	0.22	0.35	4.71	3.71	0.26	0.25	0.10	0.14
Amplitude	0.07	0.10	0.10	0.10	0.07	0.07	0.05	0.06
Sleep percentage (%)	4.92	5.88	6.59	4.81	4.27	4.51	3.01	3.83

Sleep index / Cluster	4-2-1	4-2-2	4-2-3	4-2-4	4-2-5	4-2-6	4-2-7	4-3	5
ST long MN (h)	0.73	0.81	0.50	0.26	0.37	0.40	0.49	2.27	0.75
ST long SD (h)	0.56	0.33	0.25	0.53	0.48	0.35	0.56	0.92	0.33
WT long MN (h)	0.65	0.95	0.32	0.25	0.46	0.32	0.43	1.12	0.49
ST long SD (h)	0.36	0.38	0.13	0.17	0.26	0.22	0.42	0.85	0.20
ST short MN (h)	0.45	0.46	0.29	0.03	0.10	0.20	0.15	0.56	0.00
ST short SD (h)	0.38	0.23	0.13	0.06	0.09	0.16	0.15	0.48	0.00
WT short MN (h)	0.30	0.22	0.09	0.01	0.06	0.14	0.04	0.27	0.00
ST short SD (h)	0.25	0.17	0.12	0.03	0.06	0.16	0.05	0.28	0.00
Long sleep window length MN (h)	1.04	0.77	0.46	0.33	0.67	0.48	0.62	4.44	0.81
Long sleep window length SD (h)	0.73	0.59	0.28	0.62	0.50	0.39	0.71	4.00	0.40
Long sleep window # MN	0.00	0.00	0.00	0.00	0.00	0.00	0.00	0.19	0.00
Long sleep window # SD	0.00	0.00	0.00	0.00	0.00	0.00	0.00	0.15	0.00
Short sleep window length MN (h)	0.40	0.32	0.17	0.17	0.21	0.39	0.14	0.55	0.00
Short sleep window length SD (h)	0.33	0.21	0.16	0.01	0.14	0.20	0.17	0.43	0.00
Short sleep window # MN	0.55	0.49	0.57	0.12	0.23	0.38	0.25	0.67	0.00
Short sleep window # SD	0.30	0.30	0.28	0.24	0.14	0.21	0.17	0.41	0.00
Phase MN (h)	1.88	0.90	1.24	1.62	0.85	1.29	0.80	2.24	0.78
Phase SD (h)	0.90	0.34	0.30	0.79	0.34	0.52	0.28	1.18	0.27
Period (h)	0.91	0.43	0.35	0.53	0.74	0.96	0.22	2.00	0.13
Amplitude	0.05	0.05	0.03	0.02	0.03	0.03	0.03	0.12	0.05
Sleep percentage (%)	3.22	2.41	1.63	1.10	1.51	1.99	1.63	8.35	3.13

Table S7. Means of sleep indexes

Sleep index / Cluster	1	2a	2b	3a	3b	4a	4b	5
ST long MN (h)	7.33	5.16	0.00	5.15	4.73	6.37	6.88	7.08
ST long SD (h)	1.29	2.50	0.00	2.92	2.65	1.46	0.95	0.70
WT long MN (h)	1.78	1.20	0.00	1.26	0.79	1.26	1.15	1.02
ST long SD (h)	0.88	0.79	0.00	1.00	0.58	0.63	0.48	0.34
ST short MN (h)	0.67	1.33	2.23	1.78	1.53	0.58	0.42	0.00
ST short SD (h)	0.60	1.09	0.73	1.83	1.75	0.57	0.44	0.00
WT short MN (h)	0.43	0.49	1.18	0.64	0.41	0.26	0.22	0.00
ST short SD (h)	0.40	0.44	0.58	0.58	0.41	0.28	0.25	0.00
Long sleep window length MN (h)	7.53	7.00	0.00	6.51	7.13	7.78	8.03	8.09
Long sleep window length SD (h)	2.33	1.94	0.00	1.94	1.26	2.06	1.20	0.80
Long sleep window # MN	1.21	0.91	0.00	0.98	0.77	0.99	1.00	1.00
Long sleep window # SD	0.40	0.46	0.00	0.63	0.40	0.07	0.00	0.00
Short sleep window length MN (h)	0.91	1.21	1.05	1.38	1.46	0.84	0.76	0.00
Short sleep window length SD (h)	0.61	0.82	0.80	1.02	1.01	0.52	0.43	0.00
Short sleep window # MN	1.14	1.50	3.29	1.84	1.45	0.94	0.82	0.00
Short sleep window # SD	0.78	0.95	1.03	1.23	1.07	0.76	0.66	0.00
Phase MN (h)	15.20	12.92	15.34	15.38	15.37	16.46	15.20	15.22
Phase SD (h)	0.73	6.46	2.63	0.86	0.67	1.97	0.56	0.48
Period (h)	23.94	23.99	23.79	23.92	23.94	25.19	23.93	23.93
Amplitude	0.66	0.57	0.28	0.61	0.57	0.60	0.62	0.61
Sleep percentage (%)	33.90	27.57	10.20	29.50	26.52	29.41	30.84	29.68

Table S8. Standard deviations of sleep indexes

Sleep index / Cluster	1	2a	2b	3a	3b	4a	4b	5
ST long MN (h)	1.16	1.84	0.00	1.16	1.23	1.26	0.93	0.75
ST long SD (h)	0.59	1.01	0.00	0.55	0.50	0.82	0.45	0.33
WT long MN (h)	0.93	0.90	0.00	0.68	0.51	0.81	0.60	0.49
ST long SD (h)	0.48	0.52	0.00	0.48	0.36	0.52	0.29	0.20
ST short MN (h)	0.52	0.92	1.18	0.70	0.69	0.48	0.37	0.00
ST short SD (h)	0.40	0.57	0.31	0.47	0.47	0.41	0.33	0.00
WT short MN (h)	0.40	0.47	0.56	0.46	0.35	0.27	0.25	0.00
ST short SD (h)	0.31	0.32	0.32	0.33	0.27	0.24	0.23	0.00
Long sleep window length MN (h)	1.23	1.53	0.00	1.12	1.15	2.09	1.07	0.81
Long sleep window length SD (h)	0.75	1.28	0.00	0.76	0.63	2.13	0.58	0.40
Long sleep window # MN	0.10	0.27	0.00	0.12	0.12	0.08	0.00	0.00
Long sleep window # SD	0.06	0.22	0.00	0.07	0.05	0.17	0.00	0.00
Short sleep window length MN (h)	0.48	0.49	0.35	0.35	0.49	0.46	0.46	0.00
Short sleep window length SD (h)	0.38	0.34	0.20	0.20	0.26	0.38	0.36	0.00
Short sleep window # MN	0.70	0.90	1.02	0.80	0.74	0.59	0.55	0.00
Short sleep window # SD	0.35	0.37	0.44	0.40	0.34	0.34	0.27	0.00
Phase MN (h)	1.16	3.19	2.11	1.28	1.06	1.81	0.89	0.78
Phase SD (h)	0.47	1.90	2.26	0.61	0.48	0.93	0.32	0.27
Period (h)	0.23	4.71	3.71	0.25	0.26	1.09	0.14	0.13
Amplitude	0.07	0.10	0.10	0.07	0.07	0.08	0.06	0.05
Sleep percentage (%)	4.97	6.59	4.81	4.51	4.27	4.98	3.83	3.13

Table S9. Means of sleep indexes (outliers)

Sleep index / Cluster	3b-1	3b-2	4b-1	4b-2	4b-3	4b-4	4b-5	4b-6
ST long MN (h)	4.05	2.80	8.77	6.68	6.61	5.14	6.40	6.52
ST long SD (h)	2.72	1.82	1.05	1.07	1.07	0.88	1.06	1.11
WT long MN (h)	0.53	1.66	1.70	1.24	1.02	2.85	0.25	1.10
ST long SD (h)	0.45	1.21	0.76	0.54	0.44	0.79	0.11	0.44
ST short MN (h)	2.09	1.39	0.59	0.56	0.44	0.44	0.47	0.55
ST short SD (h)	1.85	1.24	0.50	0.53	0.46	0.42	0.50	0.54
WT short MN (h)	0.47	0.84	0.39	0.26	0.20	0.31	0.17	0.24
ST short SD (h)	0.40	0.77	0.38	0.28	0.22	0.33	0.20	0.26
Long sleep window length MN (h)	6.60	6.34	10.47	7.91	7.63	7.99	6.66	7.62
Long sleep window length SD (h)	1.24	1.18	1.58	1.36	1.29	1.31	1.10	1.32
Long sleep window # MN	0.69	0.69	1.00	1.00	1.00	1.00	1.00	1.00
Long sleep window # SD	0.43	0.43	0.00	0.00	0.00	0.00	0.00	0.00
Short sleep window length MN (h)	1.47	1.35	0.84	0.87	0.79	0.77	0.79	0.84
Short sleep window length SD (h)	1.00	0.97	0.54	0.52	0.40	0.46	0.44	0.50
Short sleep window # MN	1.82	1.78	1.12	0.93	0.82	0.96	0.80	0.92
Short sleep window # SD	1.11	1.09	0.75	0.69	0.65	0.71	0.65	0.70
Phase MN (h)	15.38	15.88	14.93	12.94	15.38	15.43	15.26	17.92
Phase SD (h)	0.77	0.92	0.58	0.67	1.21	0.67	0.53	0.86
Period (h)	23.95	23.96	23.94	23.93	23.18	23.90	23.95	23.92
Amplitude	0.57	0.41	0.75	0.62	0.61	0.48	0.62	0.61
Sleep percentage (%)	26.06	18.13	39.60	30.61	29.80	23.79	28.98	29.89

Table S10. Standard deviations of sleep indexes (outliers)

Sleep index / Cluster	3b-1	3b-2	4b-1	4b-2	4b-3	4b-4	4b-5	4b-6
ST long MN (h)	1.28	1.10	0.68	0.90	0.83	1.08	0.75	0.95
ST long SD (h)	0.53	0.47	0.55	0.51	0.46	0.37	0.42	0.51
WT long MN (h)	0.41	0.70	0.91	0.49	0.47	0.72	0.13	0.55
ST long SD (h)	0.33	0.40	0.45	0.28	0.27	0.37	0.07	0.26
ST short MN (h)	0.87	0.66	0.49	0.43	0.35	0.37	0.39	0.47
ST short SD (h)	0.51	0.39	0.35	0.35	0.33	0.28	0.34	0.38
WT short MN (h)	0.36	0.55	0.36	0.24	0.21	0.30	0.19	0.26
ST short SD (h)	0.25	0.33	0.28	0.23	0.21	0.26	0.20	0.24
Long sleep window length MN (h)	1.22	1.13	1.12	1.05	0.97	1.47	0.77	1.09
Long sleep window length SD (h)	0.69	0.67	0.85	0.64	0.57	0.59	0.44	0.62
Long sleep window # MN	0.16	0.18	0.00	0.02	0.00	0.00	0.00	0.00
Long sleep window # SD	0.05	0.06	0.01	0.03	0.00	0.01	0.00	0.00
Short sleep window length MN (h)	0.43	0.45	0.43	0.47	0.48	0.44	0.42	0.47
Short sleep window length SD (h)	0.23	0.25	0.35	0.37	0.35	0.36	0.35	0.37
Short sleep window # MN	0.82	0.91	0.66	0.55	0.47	0.64	0.48	0.60
Short sleep window # SD	0.36	0.35	0.31	0.28	0.29	0.31	0.27	0.29
Phase MN (h)	1.37	1.36	0.93	1.04	0.96	1.08	0.66	0.81
Phase SD (h)	0.61	0.60	0.32	0.42	0.57	0.37	0.32	0.57
Period (h)	0.42	0.40	0.12	0.16	0.31	0.18	0.13	0.17
Amplitude	0.06	0.07	0.05	0.05	0.05	0.06	0.04	0.06
Sleep percentage (%)	4.10	3.87	2.64	3.42	3.30	4.55	2.84	3.90

Table S11. Summary of insomnia-like clusters

Cluster	Sleep duration	Fragmented	Long-term awake	Short-term awake
1	↑	↑↑	↑	↑
3a	-	↑↑↑	↑↑	↑
3b	↓	↑	↑	-
3b-1	↓	↑↑	↑↑↑	↓
3b-2	↓↓↓	↑↑	↑	↑↑
4b-4	↓↓	-	↓	↑↑↑
4b-5	-	-	↑↑	↓↓

The arrows represent how far each sleep-related indicator in the upper row is from the mean of the whole dataset in terms of seven levels (from ↓↓↓ to ↑↑↑). “Fragmented” parameter represents the summation of the number of long sleep window MN and short sleep window MN. “Long-term midawake” represents how midawake duration increases if the threshold used to connect sleep episodes to make sleep windows is 120 minutes. “Short-term midawake” represents the summation of WT long MN and WT short MN. The actual values are shown in Table S12.

Table S12. Values of insomnia-like clusters

Cluster	Sleep duration (%)	Fragmented	Long-term awake (min)	Short-term awake (h)
1	33.90	2.35	1.08	2.21
3a	29.50	2.82	1.91	1.90
3b	26.52	2.21	1.60	1.20
3b-1	26.06	2.51	2.44	1.00
3b-2	18.13	2.47	1.15	2.50
4b-4	23.79	1.96	0.47	3.15
4b-5	28.98	1.80	1.74	0.43
all mean	30.33	1.87	0.79	1.38
all sd	4.34	0.65	8.48	0.77
